# Genetic Characterization of African Swine Fever Italian Clusters in the 2022–2023 Epidemic Wave by a Multi-Gene Approach

**DOI:** 10.3390/v16081185

**Published:** 2024-07-24

**Authors:** Monica Giammarioli, Claudia Torresi, Roberta Biccheri, Cesare Cammà, Maurilia Marcacci, Alessandro Dondo, Elisabetta Razzuoli, Giovanna Fusco, Francesco Casalinuovo, Maria Teresa Scicluna, Silvia Dei Giudici, Ana Maria Moreno Martin, Elisabetta Rossi, Cristina Casciari, Michela Pela, Carmen Iscaro, Carmina Gallardo, Gaia Marocco, Mario Orrico, Francesco Feliziani

**Affiliations:** 1 National Reference Laboratory (NRL) for Swine Fever, Istituto Zooprofilattico Sperimentale dell’Umbria e delle Marche “Togo Rosati”, 06126 Perugia, Italy; c.torresi@izsum.it (C.T.); r.biccheri@izsum.it (R.B.); e.rossi@izsum.it (E.R.); c.casciari@izsum.it (C.C.); m.pela@izsum.it (M.P.); c.iscaro@izsum.it (C.I.); gaia.marocco@studenti.unipg.it (G.M.); f.feliziani@izsum.it (F.F.); 2 National Reference Center for Whole Genome Sequencing of Microbial Pathogens, Database and Bioinformatic Analysis, Istituto Zooprofilattico Sperimentale dell’Abruzzo e del Molise “G. Caporale”, 64100 Teramo, Italy; c.camma@izs.it (C.C.); m.marcacci@izs.it (M.M.); 3 Istituto Zooprofilattico Sperimentale Piemonte, Liguria e Valle d’Aosta “I. Altara”, 10154 Turin, Italy; alessandro.dondo@izsto.it (A.D.); elisabetta.razzuoli@izsto.it (E.R.); 4 Istituto Zooprofilattico Sperimentale del Mezzogiorno, Portici, 80055 Napoli, Italy; giovanna.fusco@izsmportici.it (G.F.); f.casalinuovo@izsmportici.it (F.C.); 5 Istituto Zooprofilattico Sperimentale del Lazio e della Toscana “M. Aleandri”, 00178 Rome, Italy; teresa.scicluna@izslt.it; 6 Istituto Zooprofilattico Sperimentale della Sardegna “G. Pegreffi”, 07100 Sassari, Italy; silvia.deigiudici@izs-sardegna.it; 7 Istituto Zooprofilattico Sperimentale della Lombardia e della Emilia Romagna “Bruno Ubertini”, 25124 Brescia, Italy; anamaria.morenomartin@izsler.it; 8 European Union Reference Laboratory for African Swine Fever (EURL), Centro de Investigación en Sanidad Animal (CISA), Instituto Nacional de Investigación y Tecnología Agraria y Alimentaria (INIA), Consejo Superior de Investigaciones Científicas (CSIC), Valdeolmos, 28130 Madrid, Spain; gallardo@inia.csic.es; 9 Veterinary Practitioner, 87040 Cosenza, Italy; mario.orrico90@gmail.com

**Keywords:** African swine fever virus, genotype II, multi-gene approach, genetic groups, Italian clusters

## Abstract

The first report of African swine fever virus (ASFV) genotype II in Italy in 2022 marked the beginning of a significant invasion in at least eight Italian regions with different infection clusters. In this study, we used the multi-gene approach to investigate the epidemiological associations between ASFV strains causing cases and outbreaks in wild boar and pigs in Italy from January 2022 to the end of 2023. Our results confirm that all the tested ASFV-positive Italian samples belonged to genotype II and show high homology with genotype II ASFV sequences previously collected in Eurasian countries. Molecular characterization revealed the presence of four genetic groups in Italy. The majority of African swine fever (ASF) samples analyzed in the current study (72%) belonged to genetic group 3, which was the most representative in Europe. The results also provide evidence of the prevalence of genetic group 19 (15.9%). In addition, we identified new putative genetic groups, genetic group 25 (9.1%) and genetic group 26 (3.0%), which have never been described before. This is the first detailed report on the molecular characterization of more than 130 ASFV strains circulating in Italy.

## 1. Introduction

The first report of African swine fever virus (ASFV) genotype II in Georgia in 2007 marked the beginning of a major worldwide invasion, with more than 50 infected countries. Prior to the spread of genotype II from Africa to Georgia in 2007 [1], outbreaks of ASFV genotype II were reported in Zambia in 1993, Mozambique and Madagascar in 1998 [2] and on the island of Mauritius in the Indian Ocean in 2007/2008 [3]. After 2007, outbreaks associated with ASFV genotype II were documented in Tanzania (2010), coinciding with outbreaks in Malawi, Zimbabwe (2015) and South Africa (2019) [4]. Since its introduction into the northern hemisphere through Georgia, the disease has spread to the Transcaucasian region, certain regions of the Russian Federation and Eastern Europe, including at least seventeen European countries [5,6]. In 2018, ASFV reappeared in northwestern Europe (Belgium), presenting exclusively within the wild boar (*Sus scrofa*) population, and was first detected in the People’s Republic of China, where catastrophic outbreaks occurred [7]. In 2019, ASFV was detected in several countries bordering China, including Cambodia, Mongolia, Vietnam, North Korea, Myanmar, Lao People’s Democratic Republic, Timor-Leste, the Philippines, South Korea and Indonesia [7,8]. In 2020 and 2021, African swine fever (ASF) cases in wild boar were reported in Germany, the Dominican Republic and Haiti [9,10]. The disease has spread rapidly to 15 other Asian and Pacific countries [11]. In early 2020, it was also reported in two northeastern states of India [12]. The country most recently impacted is Albania [13]. 

In January 2022, cases of ASF caused by genotype II were reported in northwestern Italy and in Sweden in September 2023 [14,15,16]. In Italy, a second infection was reported in May 2022 in the Lazio region (in the municipality of Rome, where the infection is still present) [17]. In northwestern Italy, the virus continued to spread in Liguria and Piedmont until 2023, causing several positive cases in wild boar. From this cluster, the infection reached Lombardy, first affecting the wild population (province of Pavia, June 2023) and then domestic pigs in August 2023. Due to the proximity to the infected areas of Lombardy, the wild population of Emilia Romagna was also affected in November 2023. In May 2023, ASF genotype II continued to spread in southern Italy. In the Calabria region, wild boar and domestic pigs were affected, while in the Campania region, only the wild population has been involved.

In September 2023, ASFV genotype II was detected on the island of Sardinia, as a consequence of outbreaks in Pavia province. Pig’s meat imported from Lombardy was suspected to be the cause of this outbreak. Strict quarantine and slaughter measures limited the spread of the disease, and the outbreaks were successfully eradicated [18].

ASFV is a complex cytoplasmic-replicating icosahedral virus. It has been placed in a newly created family, the *Asfarviridae*. This unique virus belongs to the monotypic genus Asfiviruses and is currently the only known double-stranded deoxyribonucleic acid (dsDNA) virus transmitted by arthropods (Arbovirus) [19,20]. The genome size of ASFV ranges from 170 to 193 kilobase pairs (kbp) [21]. Although the central region of the genome is conserved, there are a large number of differences at both the left and right ends of the genome, which are approximately 40 kbp and 10–12 kbp long, respectively [22].

The left variable and right variable regions of the genome consist of copies of different multigene families (MGF) and several other genes [20]. Differences in the number of short tandem repeat sequences (TRS) present at loci within the coding regions, and the intragenic regions also contribute to the variation in genome size of the virus [21]. The ASFV genome contains between 151 and 167 predicted open reading frames (ORF) [21,23], closely arranged in both DNA strands, with no clear bias towards coding genes on one of the strands throughout the genome [21]. 

ASFVs are classified into 24 genotypes (I-XXIV) based on the partial sequences of the C-terminal region of the B646L/p72 gene, which encodes the capsid protein p72 [24,25,26,27,28]. Of the 24 genotypes, genotype II is the most common and has a high degree of sequence identity in the gene markers commonly used for genotype assignment and within-genotype resolution [2,28]. The high degree of homogeneity and the limited number of sequences for the African genotype II viruses prior to the 2007 excursion from Africa to the Caucasus have hampered the identification of the most likely origin of the virus introduced into Georgia in 2007. According to the data regarding B646L/p72 and the central variable region (CVR) located within the B602L gene in East Africa and Madagascar during the period of the excursion [2,25,29], East Africa was identified as the most likely region of origin [1].

Genotyping of ASFV during outbreaks is important to study the origin of the virus and to rapidly distinguish between closely related strains [30] to advance our knowledge of viral evolution and molecular epidemiology. The most common approach for genotyping ASFV during outbreaks is based on analysis of the C-terminal end of the B646L/p72 [24]. Sequencing of the E183L gene, which encodes the p54 protein, and the CVR within the B602L gene are also used to distinguish between geographically and temporally linked p72 genotypes [2,22,31,32,33,34]. The B602L gene consists of amino acid tetramers that vary in number and type [34,35]. The reasons for the high degree of variation in the B602L gene are unclear. However, the gene is thought to play a role as a chaperone in the assembly of the capsid protein p72 into virions, while it is not integrated into the virions [36]. Although the value of CVR has been demonstrated, particularly for genotype I viruses, there are two major drawbacks to its use: (i) the analysis of this gene region is complex and not readily standardized between laboratories, and (ii) it is restricted to analyses within the genotype [2,37].

Other approaches exist to assess the evolutionary differences between ASFV strains, particularly the evolutionary connections of strains in the same region, as the assessment of p30, a structural protein encoded by the CP204L gene, which plays a crucial role in virus emergence [38,39] and has been shown to be able to discriminate between closely related viruses [1,40,41,42]: EP402R, which encodes CD2v, plays a role in ASFV hemadsorption and is important for serotyping and genotyping [42]; TRS within the intergenic region (IGR) between I73R and I329L (ECO1) [41,42]; the thymidine kinase (TK) gene [43]; the J268L, Bt/Sj, KP86R [34] and O174L genes [44]; and the C315R/C147L region [40]. Seven molecular markers, defined as “additional”, have been proposed in recent studies [37] based on whole genome sequences, and are capable of sub-genotype ASF genotype II.

We used a multi-gene approach with six essential molecular markers to investigate the epidemiological associations between ASFV strains causing outbreaks in wild boar and pigs in mainland Italy between January 2022 and the end of 2023. The study started with an initial analysis of the TRS in the CVR, in the IGR between I73R and I329L (ECO1 region) and in the O174L gene, and with the sequencing of the SNP in the K145R gene. For additional discrimination, two other PCRs were used to amplify molecular markers characterized by the presence of TRS: the IGR between the 9R and 10R genes of multigene family 505 (MGF) and the IGR between the I329L and I215L genes (ECO2 region) [37,45]. This is the first detailed report on the molecular characterization of more than 130 ASFV strains circulating in Italy during the 2022–2023 epidemic wave.

## 2. Materials and Methods

### 2.1. Specimens

A total of 132 positive samples were selected from the virus repository of the National Reference Laboratory (NRL) for Swine Fever of Istituto Zooprofilattico Sperimentale Umbria and Marche “Togo Rosati” (IZSUM) in Italy. 

The comprehensive features of ASFVs, along with the outcomes acquired for each virus, are presented in Supplementary Table S1.

**Figure 1:**
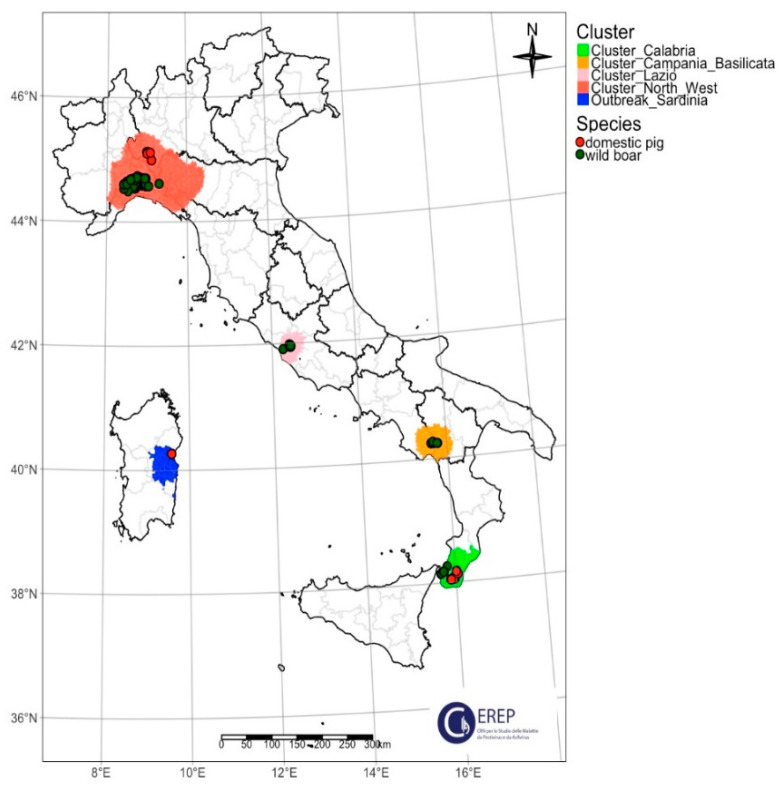
Italian ASF infected areas. The map represents the geographical location of the analyzed samples of wild boar and domestic pigs collected in different infected areas. The northwestern cluster included four regions (Piedmont, Liguria, Lombardy and Emilia-Romagna); the Lazio cluster comprised only the municipality of Rome; the Campania cluster involved two regions (Campania and Basilicata); the Calabria cluster and Sardinia outbreak concerned only Calabria and Sardinia, respectively. The red and green dots represent the samples of domestic and wild boar analyzed in the study.

The samples were collected according to national surveillance/eradication plan or for confirmation of suspicious cases from infected Italian areas. In particular, 103 samples of wild boar and 29 of domestic pigs were selected for the study. The samples chosen have a geographical and temporal representation of all the infected territories (Table 1). The samples were collected from 7 January 2022 (first Italian case notified in Alessandria province, Piedmont region) to 16 November 2023 (first case in Piacenza province, Emilia Romagna region). The current Italian epidemiological scenario, with four ASF clusters on the mainland and one outbreak on Sardinia Island, is shown in Figure 1.

At the beginning of 2022, the first case of ASFV genotype II was reported in wild boar in northwestern Italy [14]. This area represents the cluster of the northwest and includes the regions of Piedmont, Liguria, Lombardy and Emilia-Romagna. Samples from all the infected provinces were analyzed: Alessandria (Piedmont region), Genoa and Savona (Liguria region), Pavia (Lombardy region) and Piacenza (Emilia Romagna region). The second cluster is located in the Lazio region, more precisely, within the city limits of Rome. The third cluster is located in the south of the Italian peninsula and includes the region of Calabria, where the infection affects wild boar and domestic pigs in the province of Reggio Calabria. The last cluster detected is in the Campania region, a central-southern Italian region where one province, Salerno, was affected. In Sardinia, a single-point infection was reported (province of Nuoro), probably due to wide jumping of the virus from the infected areas of the peninsula [18].

The clinical samples (103 spleen, 26 bone marrow, 3 kidney) were homogenized to obtain a 10% *w*/*v* suspension that was centrifuged at 1200× *g* for 10 minutes. The DNA was extracted using a High Pure PCR Template Preparation Kit (Roche Diagnostics GmbH, Roche Applied Science, Mannheim, Germany) according to the manufacturer’s instructions and tested for the presence of porcine beta-actin [46] and for ASFV by real-time PCR [47,48].

### 2.2. Molecular Marker Analysis: Conventional PCR, Sanger Sequencing and Phylogenetic Analysis

The samples were genotyped by partial Sanger sequencing of a fragment of the B646L/p72 gene as previously described [24]. The B646L/p72 sequence dataset was analyzed, and the nucleotide sequences were aligned to the published ASFV reference strains retrieved from GenBank using Clustal X.2 [49] and manually cured using BioEdit software (version 7.0) [50]. The phylogenetic tree was inferred using the maximum likelihood (ML) method. An ML analysis was performed in MEGA 11 [51]. Cluster robustness was evaluated by performing 10,000 bootstrap replications, and branches with bootstrap values greater than 70% were clustered. A consensus tree was displayed and edited using iTOL v.6 [52].

**Figure 2:**
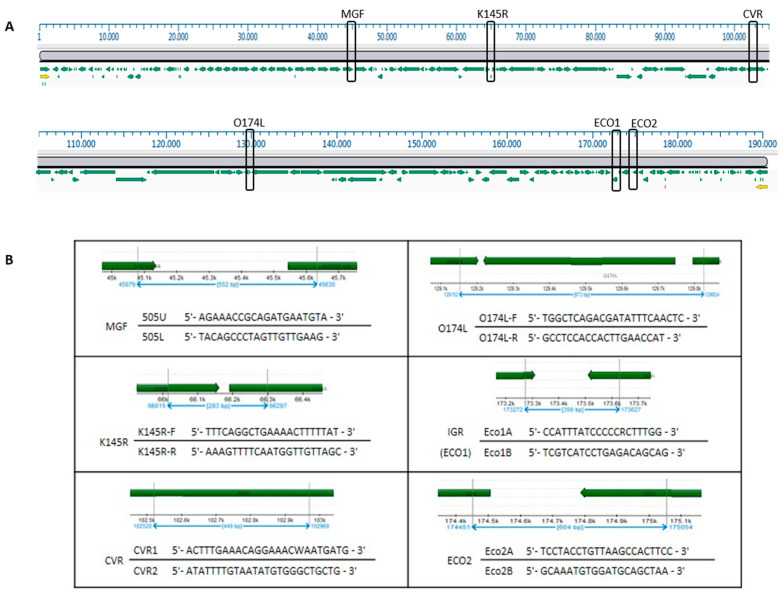
The genomic regions analyzed in this study. (**A**): Diagram showing the location of the molecular genomic regions in reference to Georgia ASFV 2007/1 (FR682468). (**B**): Size of the amplified targets, nucleotide sequences and position of the primers.

Molecular characterization was based on the multi-gene approach proposed by Gallardo and collaborators [45]. The genomic regions analyzed in the study were CVR within the B602L gene, the intergenic region between the I73R and I329L genes (IGR-ECO1), the complete O174L gene, the partial sequence of the K145R gene, the intergenic region between MGF 505-9R and MGF 505-10R (MGF) and the intergenic region between the I329L and I215L genes (ECO2). The location of the molecular markers in reference to Georgia ASFV 2007/1, the nucleotide sequences of the primers and the corresponding position are shown in Figure 2.

Six independent PCR reactions were performed using the published primers [45] and AmpliTaq Gold DNA Polymerase (Thermo Fisher Scientific, Waltham, MA, USA) according to the manufacturer’s instructions. The cycling conditions were identical for the six molecular markers, with an anneal temperature of 55 °C. After gel electrophoresis, the PCR products were purified using an ExS-PURE PCR cleanup Kit (NimaGen BV, Nijmegen, The Netherlands). Both the sense and antisense strands were sequenced by Sanger sequencing by performing three independent reactions for each isolate using a BrilliantDye v1.1 cycle sequencing Kit (NimaGen BV, Nijmegen, The Netherlands), and the dye terminators were removed with a DyeEx 2.0 Spin Kit (Qiagen, Hilden, Germany). The precipitated products were run on an ABI PRISM 3130 Genetic Analyzer (Thermo Fisher Scientific, Waltham, MA, USA), and the sequence data were analyzed using the SeqMan Pro^®^ program Version 15.0 (DNASTAR, Madison, WI, USA) [53]. The nucleotide sequences were aligned using Clustal X.2. [49], manual sequence editing was performed using BioEdit software (version 7.0.) [50].

The nucleotide sequences obtained are available in GenBank (the list of accession numbers is in Supplementary Table S1).

The cartographic representations were crafted utilizing the R-studio environment [54], leveraging the functionalities provided by the tmap package [55].

## 3. Results

### 3.1. Phylogenetic Analysis

A total of 132 ASF partial B646L/p72 sequences were aligned with reference strains of ASF genotypes I–XXIV. Our results confirm that the 132 positive Italian ASF samples belonged to genotype II and showed high identity (99.7–100.00%) with ASFV sequences of genotype II previously collected in Eurasian countries (Figure S1).

### 3.2. Genetic Variants of the Molecular Markers

All the samples showed 100% identity for three marker regions, namely, CVR, IGR-ECO1 and K145R (Supplementary Table S1, Supplementary Figures S2–S6).

The CVR region genotype II variant I with 10 amino acids TRS was present in all the sequenced samples and showed 100% sequence similarity with the Georgia 2007/1 strain (FR682468.2). None of them showed the SNPs already described in Poland and Lithuania.

The IGR-ECO1, which may contain a variable number of tandem repeats, as previously reported [44], remained consistent among the analyzed isolates, suggesting that they all belong to IGR-ECO1 variant II, a subtype present in over 92% of Euro-Asian ASFV strains [45].

**Figure 3:**
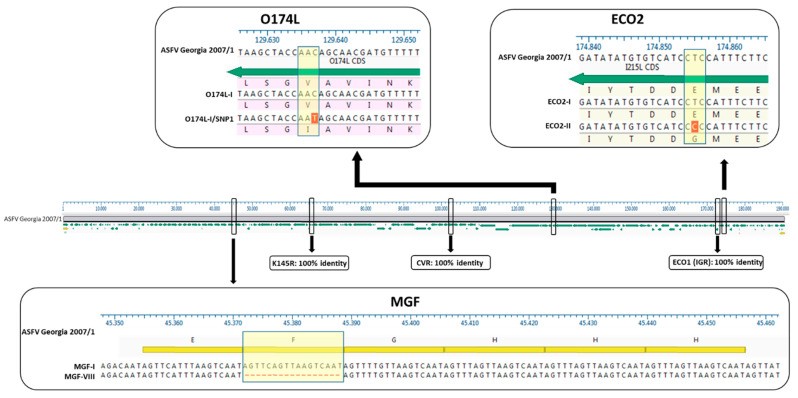
Genetic variants of the molecular markers analyzed in a multi-gene approach observed in Italian ASFV samples. All the samples showed complete identity in the three marker regions: CVR, IGR-ECO1 and K145R (100% identity). In the O174L sequence, an SNP was observed at position 109 of the gene (position 129637 in the Georgia 2007/1 strain), which introduces a new variant, O174L-I/SNP1, and leads to a change in the amino acid Val37Ile. The two already known genetic variants for ECO2 (ECO2-I and II) have been described among the ASFV strains circulating in Italy. ECO2-I and II differ by the presence of an SNP that implies an amino acid change in the I215L gene, Glu192Gly. Regarding the MGF marker, two different variants were identified in the Italian ASFV samples: MGF variant I (MGF-I) and VIII (MGF-VIII), reported here for the first time. These two variants show dissimilarities due to the absence of the F repeat sequence (AGTTCAGTTAAGTCAAT) in the second series of TRS.

Finally, the molecular marker K145R did not show any variations in the Italian samples analyzed, so only the K145R variant I has been identified at present (Figure 3).

The O174L gene was conventionally sequenced using the same panel of samples. A total of 128 out of 132 samples analyzed (96.97%) showed 100% identity with the reference strain Georgia 2007/1 and represented the O174L variant I. Variant II, with a 14-nt insertion, does not appear to be present in Italy, whereas it is present in Poland, Lithuania, Romania and Germany [56].

The remaining 4 samples (8.7% of 46; 3.03% of the 132 sample panel), consisting of wild boar samples collected in the province of Alessandria (northwest cluster), showed a point mutation in the nucleotide sequence O174L (O174L variant I-SNP1) at position 109 of the gene (position 129637 in strain Georgia 2007/1): a transition from cytosine (C ) to thymine (T), resulting in an amino acid change from valine (V) to leucine (L) at position 37 of the protein (Val37Ile, Figure 3). This SNP is described for the first time in this study, as a comparison with the sequences previously available in the database shows (March 2024). The names of the O174L-I/SNP1 isolates and the GenBank accession numbers are as follows: 8549_2284/AL/2023 (PP420393), 22700_2598/AL/2023 (PP420340), 22700_2645/AL/2023 (PP420361) and 22700_2646/AL/2023 (PP420362).

**Figure 4:**
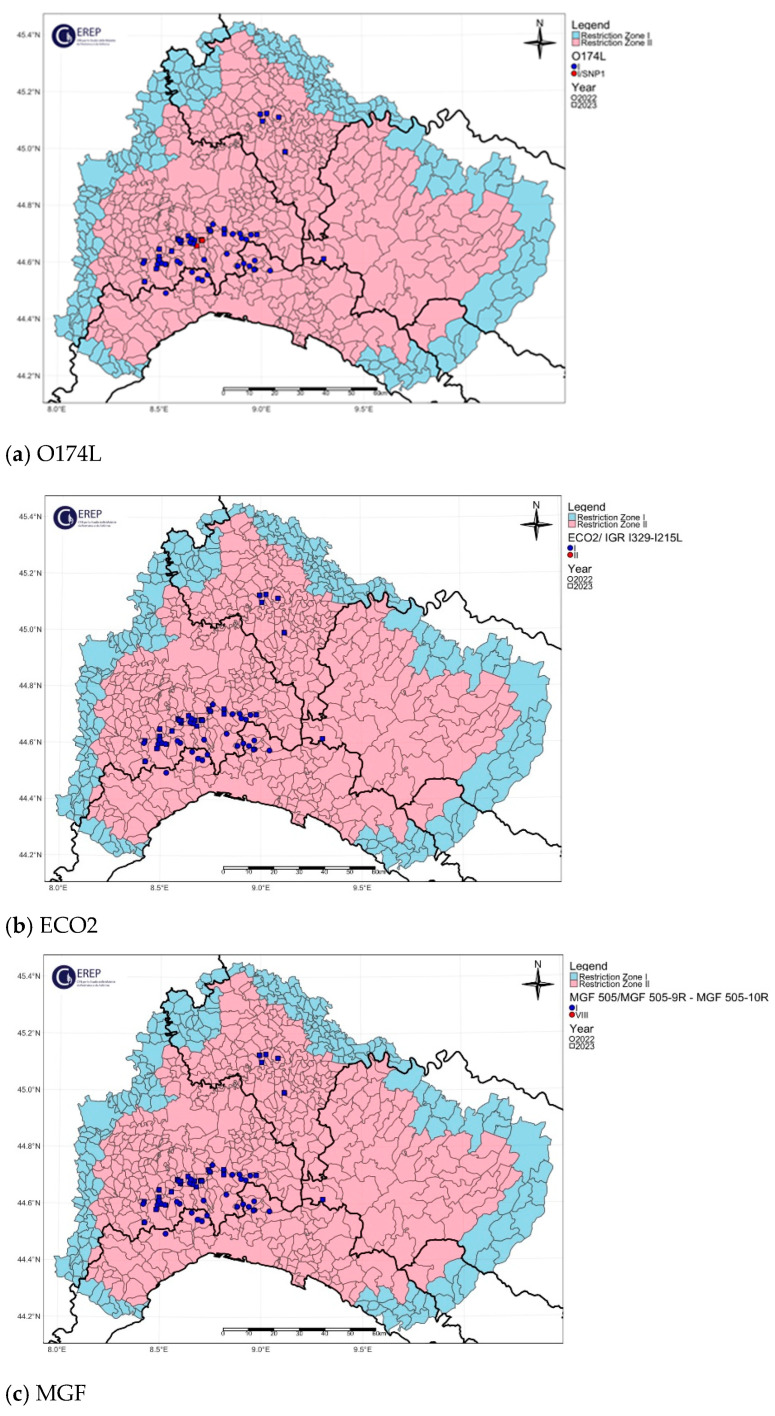
Northwestern ASF Italian cluster. Geographical distribution and identification of genetic variants of molecular markers: (**a**) O174L, (**b**) ECO2, (**c**) MGF. The pink and light blue areas represent restriction zone II and restriction zone I, respectively, as per the zoning date of 29 April 2024 (according to Commission Implementing Regulation EU 2024/1269). Round shapes indicate the year 2022; square shapes indicate the year 2023.

**Figure 5:**
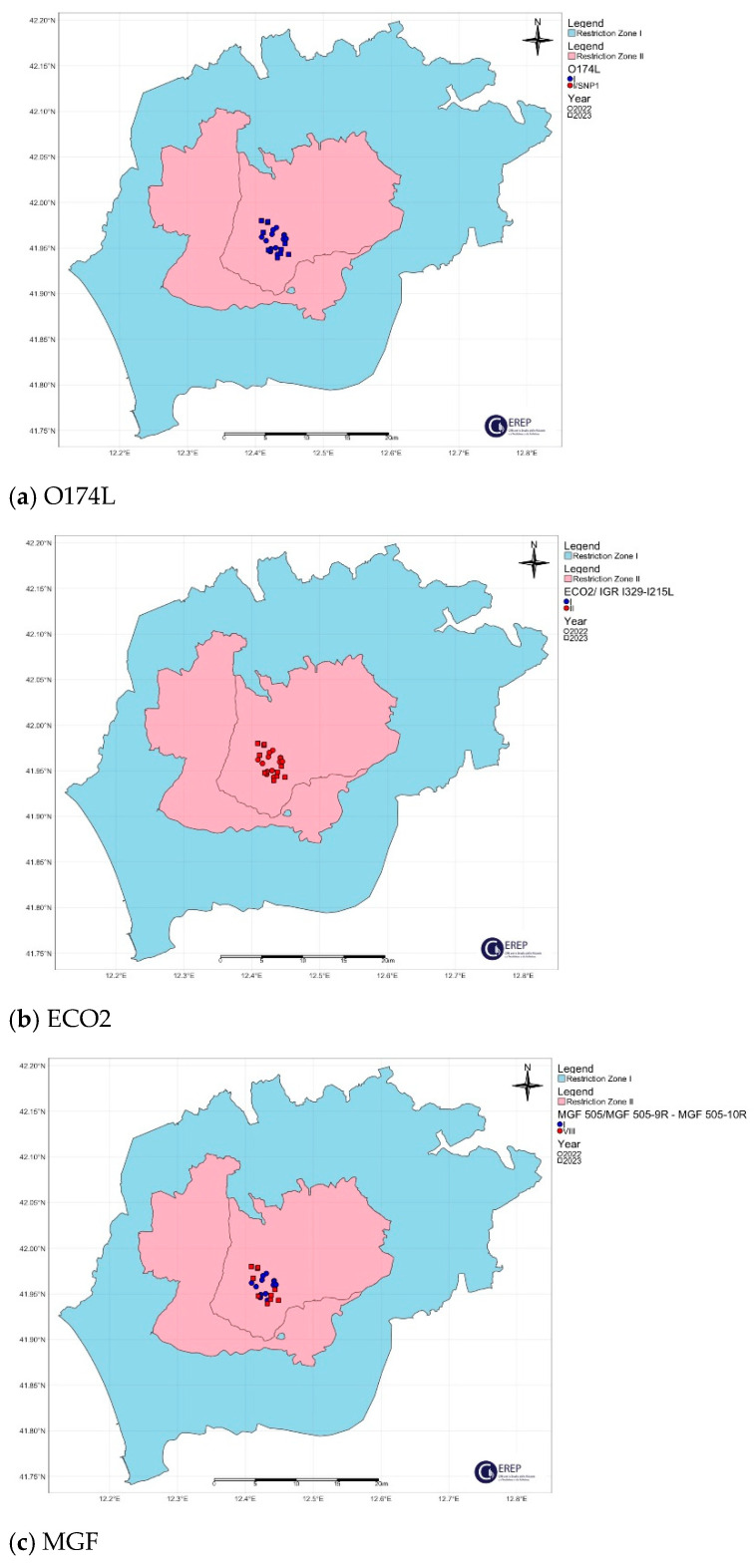
Lazio ASF Italian cluster. Geographical distribution and identification of genetic variants of molecular markers: (**a**) O174L, (**b**) ECO2, (**c**) MGF. The pink and light blue areas represent restriction zone II and restriction zone I, respectively, as per the zoning date of 29 April 2024 (according to Commission Implementing Regulation EU 2024/1269). Round shapes indicate the year 2022; square shapes indicate the year 2023.

**Figure 6:**
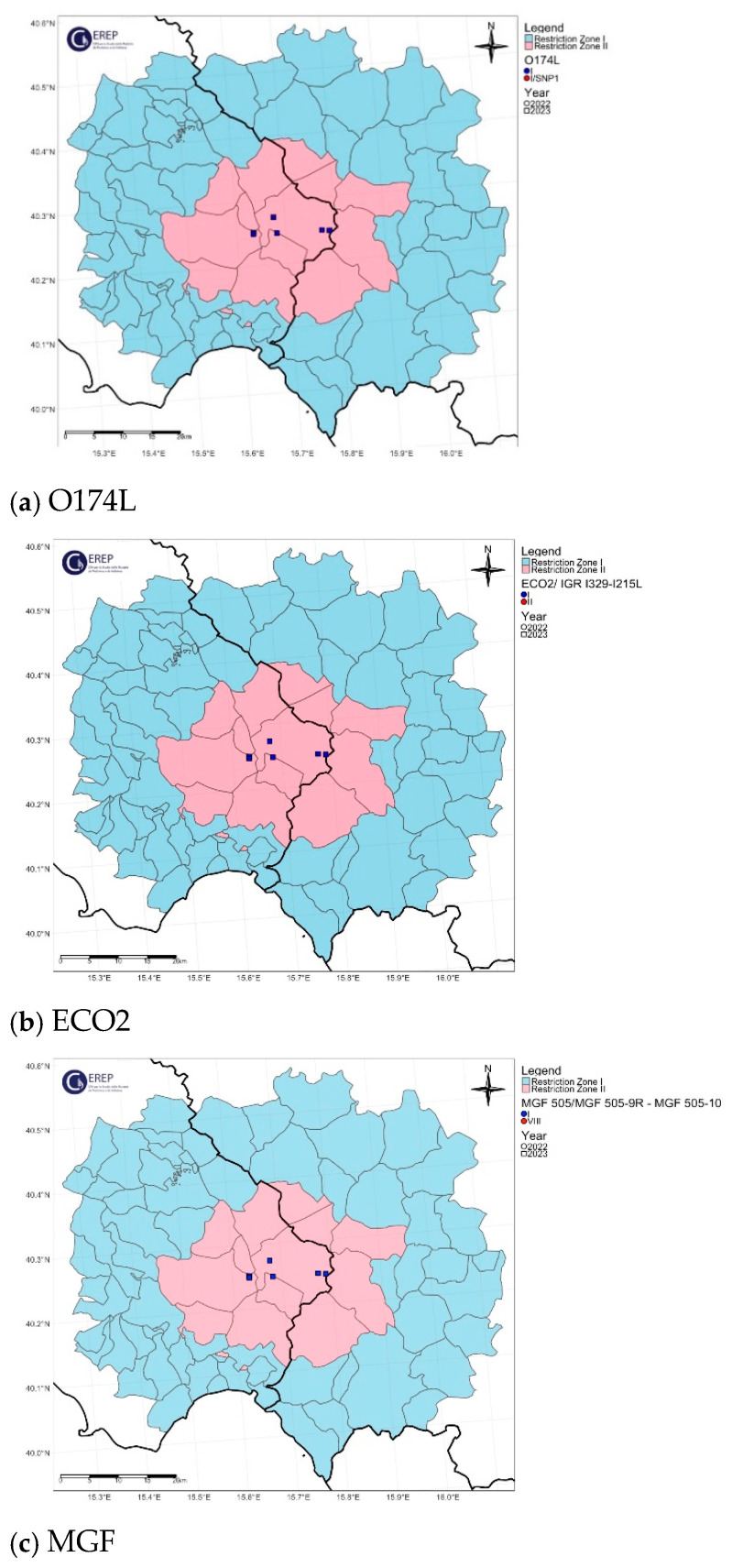
Campania ASF Italian cluster. Geographical distribution and identification of genetic variants of molecular markers: (**a**) O174L, (**b**) ECO2, (**c**) MGF. The pink and light blue areas represent restriction zone II and restriction zone I, respectively, as per the zoning date of 29 April 2024 (according to Commission Implementing Regulation EU 2024/1269). Round shapes indicate the year 2022; square shapes indicate the year 2023.

**Figure 7:**
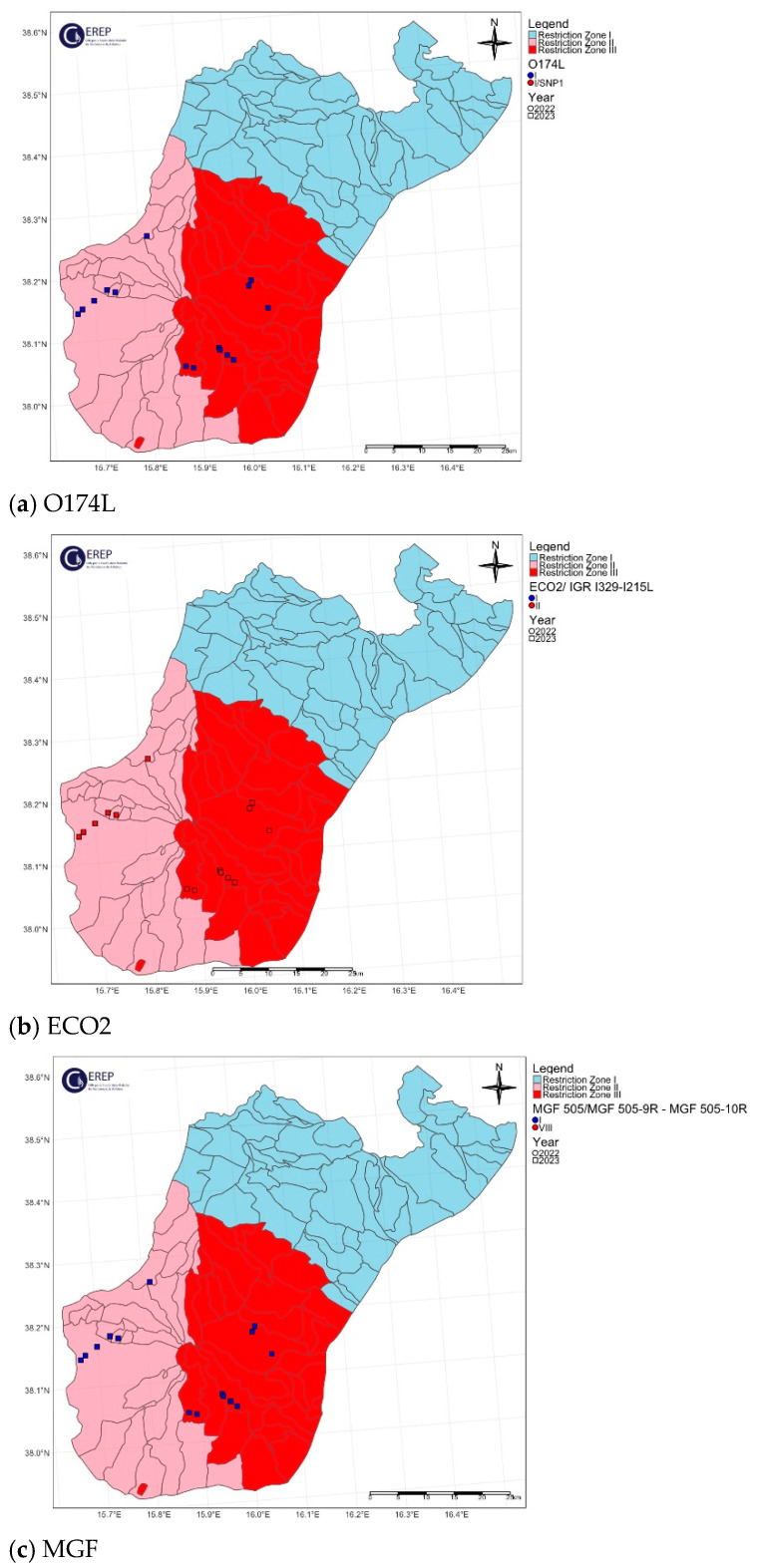
Calabria ASF Italian cluster. Geographical distribution and identification of genetic variants of molecular markers: (**a**) O174L, (**b**) ECO2, (**c**) MGF. The red, pink and light blue areas represent restriction zone III, restriction zone II and restriction zone I, respectively, as per the zoning date of 29 April 2024 (according to Commission Implementing Regulation EU 2024/1269). Round shapes indicate the year 2022; square shapes indicate the year 2023.

**Figure 8:**
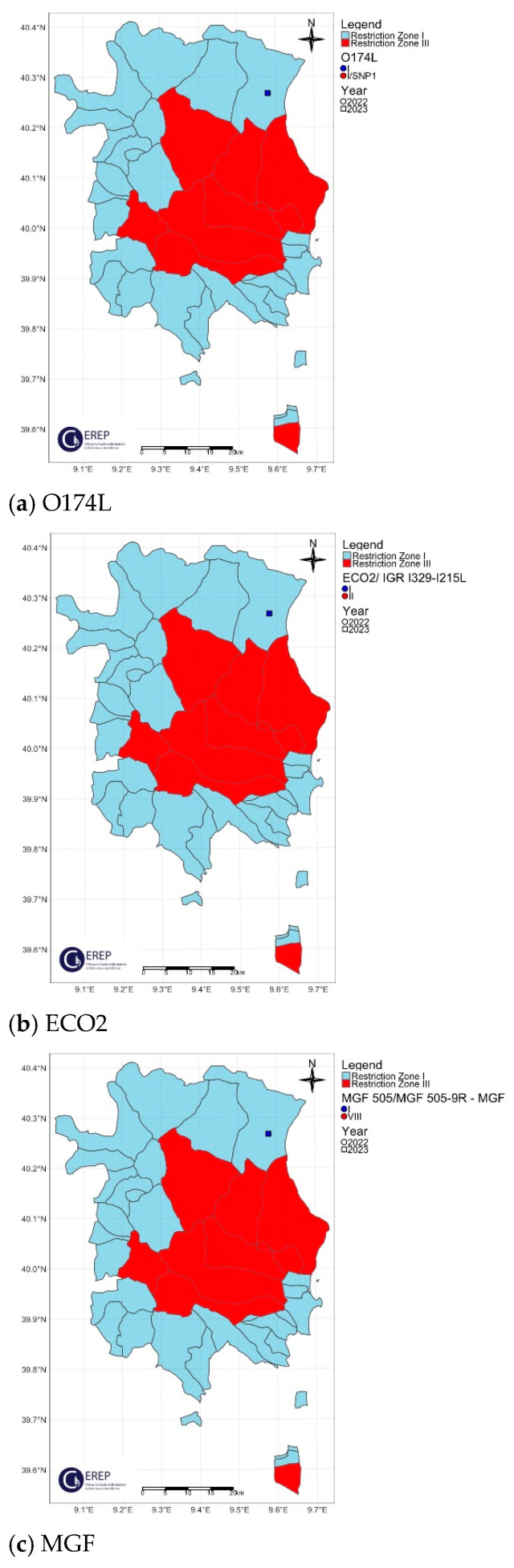
Sardinia ASF Italian outbreak. Geographical distribution and identification of genetic variants of molecular markers: (**a**) O174L, (**b**) ECO2, (**c**) MGF. The red and light blue areas represent restriction zone III and restriction zone I, respectively, as per the zoning date of 29 April 2024 (according to Commission Implementing Regulation EU 2024/1269). Round shapes indicate the year 2022; square shapes indicate the year 2023.

The spatial distribution of the O174L gene variants is shown in Figure 4a, Figure 5a, Figure 6a, Figure 7a and Figure 8a.

The two variants differ in the presence of an SNP in which C has been replaced by T, resulting in an amino acid change in the I215L gene (Glu192Gly, Figure 3). Both variants were also identified among the isolates circulating in Italy, each showing a clear geographical distribution (Figure 4b, Figure 5b, Figure 6b, Figure 7b, Figure 8b). All the samples (78 out of 132, 59.1%) collected between January 2022 and November 2023 in clusters from the northwest (provinces of Alessandria, Genoa, Savona, Pavia and Piacenza), Sardinia (province of Nuoro) and Campania (province of Salerno) belonged to ECO2 variant I (100% homology with Georgia 2007/1). The remaining 54 samples (40.9%) collected in the Lazio (province of Rome) and Calabria (province of Reggio Calabria) clusters could be assigned to ECO2 variant II, which has been described in Bulgaria, Serbia, Greece, North Macedonia and Romania, where it co-occurs with variant I [45]. 

The variability of the MGF marker region is attributed to the presence of two sets of TRS in the non-coding intergenic region between MGF 505-9R and 10R. The first set of TRS observed in all the isolates sequenced in this study followed the structure ABBCD, which is consistent with previous findings [45]. Similarly, the second set of TRS in all the infection clusters, except the cluster in Lazio (Rome municipality), matched the EFGHH pattern, resulting in a total of 11 replicates characteristic of MGF variant I, with 100% similarity to the strain from Georgia in 2007/1. The samples from the municipality of Rome showed a temporal variation: 17 wild boar and 1 domestic pig sampled in 2022 were attributable to MGF variant I and showed the same sequence of TRS observed in the rest of Italy. In 2023, the 12 samples collected, including 11 wild boar and 1 pig, showed a novel combination carried by the second set of EGHH-type TRS for the loss of 17 nt (F: AG-TTCAGTTAAGTCAAT, Figure 3). This novel combination of repeats (ABBCD-EGHH), termed MGF variant VIII, is described for the first time in this study. The strains referring to MGF-variant VIII are 21826_2300/RM/2023, 35950_2665/RM/2023, 27488_2392/RM/2023, 35950_2667/RM/2023, 35950_2670/RM/2023, 35950_2669/RM/2023, 35950_2671/RM/2023, 35950_2672/RM/2023, 35950_2674/RM/2023, 35950_2675/RM/2023, 35950_2676/RM/2023 and 33133_2529/RM/2023, and the GenBank accession numbers are listed in Table S1. 

The geographic distribution of the MGF molecular variants is shown in Figure 4c, Figure 5c, Figure 6c, Figure 7c and Figure 8c.

### 3.3. Classification of Italian ASFV Strains in Four Different Genetic Groups

**Figure 9:**
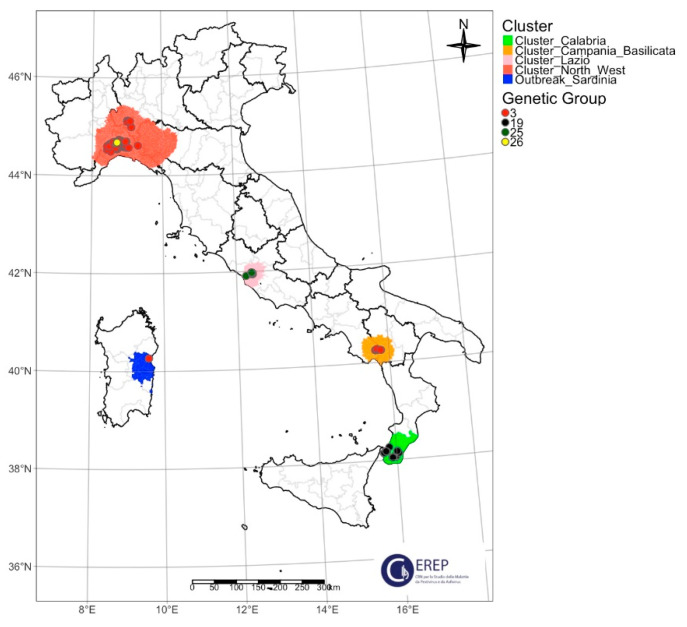
Geographical location of the ASF genetic groups identified in Italy during the 2022–2023 epidemic wave. Northwestern cluster: genetic group 3 in 2022–2023, with the exception of 4 wild boars collected in 2024 (genetic group 26). Lazio cluster: genetic group 19 in 2022, genetic group 25 in 2023, the exact geographical correspondence of the analyzed samples results in an overlap of the graphical representation of the two different genetic groups. Campania–Basilicata cluster: genetic group 3 in 2023. Calabria cluster: genetic group 19 in 2023. Sardinia outbreak: genetic group 3 in 2023.

The six molecular markers considered were evaluated globally. The combination of the variants of CVR, IGR-ECO1 I73R/I329L, O174L, K145R, MGF and ECO2 allowed the assignment of the 132 sequenced Italian strains to 4 different genetic groups, according to the classification proposed by Gallardo and collaborators [45], as shown in Table 2 and Figure 9.

The samples collected in the province of Reggio Calabria (2023) and Lazio (2022) were assigned to genetic group 19 (CVR-I, IGR-ECO1-II, O174L-I, K145R-I, MGF-I, ECO-II). In 2023, the appearance of a new variant for the MGF marker led to the definition of a new genetic group: 25 (CVR-I, IGR-II-ECO1, O174L-I, K145R-I, MGF-VIII, ECO-II), which has so far only been described in the municipality of Rome.

The samples from the northwest and the Campania–Basilicata clusters were assigned to genetic cluster 3 (CVR-I, IGR-II-ECO1, O174L-I, K145R-I, MGF-I, ECO-I), as was the one case reported in Sardinia in 2023. Exceptions can be observed in the samples collected in 2023 in the province of Alessandria: four wild boars showed an SNP carried by O174L and were consequently assigned to an additional new genetic cluster, namely, 26 (CVR-I, IGR-ECO1-II, O174L-I-SNP1, K145R-I, MGF-I, ECO-I)

Overall, the four genetic groups in Italy were distributed as follows on the basis of the samples characterized molecularly to date: 72% genetic group 3, 15.9% genetic group 19, 9.1% genetic group 25 and 3% genetic group 26.

## 4. Discussion

In the previous century, ASF was predominantly confined to the African continent because the trans-continental epidemic linked to genotype I was definitively resolved. Otherwise, genotype II is widespread worldwide, with favorable endemic trends. A transboundary ASFV genotype II introduction into mainland Italy in 2022 resulted in a slow and inexorable expansion across the northwest of the country. Other genotype II introductions of unclear origin occurred in central and southern Italy during 2022 and 2023. In September 2023, on Sardinia island, the first ASFV genotype II outbreak was detected and represented the unique certain escape of ASFV from the northwestern Italian cluster [18].

ASF is continuing to spread in peninsular Italy, involving additional new areas and outbreaks in domestic pigs. Reports also indicate a large number of cases in the wild boar population, which is consistently increasing and spreading across wide geographic areas, therefore representing a virus reservoir dangerous for the pig industry [7,57]. ASFV is highly resistant to extreme conditions, as it can survive under diverse environmental circumstances and may be transmitted through contaminated objects, contaminated food and equipment [58]. Humans can mechanically transport the ASF virus by human-mediated activities (the “human factor”) [59]. ASFV is currently confirmed in at least eight Italian regions [60].

The territories involved in the infection do not all have territorial continuity, so it is possible to hypothesize more than one introduction into the different clusters. In fact, with this work, this hypothesis could already be confirmed: the sequences of the northwest cluster belong to a different genetic group than those of the central Italy cluster (Lazio region).

While the wild boar habitat cycle comprises wild boar, the wild boar habitat and wild boar carcasses play a crucial role in sustaining and spreading the virus to domestic pigs [61]. This cycle may define prospects of new infections in areas with the presence of wild boar inhabitants [61]. Further studies are underway to verify the introductions in the different Italian clusters of infection.

On the Italian island of Sardinia, the ASFV genotype I has been endemic since 1978, although the last control measures put in place achieved a significant reduction in ASF, and the virus has been absent from circulation since April 2019. 

In Italy, two ASFV introductions in pigs have been reported in the past. The first was reported in 1967 in 28 Italian provinces. The second incursion was reported in Piedmont in March 1983 and represented the unique escape of ASFV genotype I from Sardinia; wild boar meat imprudently imported from Sardinia has been incriminated as the cause of this outbreak. Strict quarantine and slaughter measures confined the spread of the disease, and the outbreaks were successfully eradicated. Since there are no effective treatment or vaccine options available against ASF, additional information about the genetic diversity of the ASF viruses circulating in Italy must be investigated to gain insights into transmission routes to identify possible preventative mechanisms.

This study aimed to investigate the genomic patterns of ASFV clusters during 2022–2023 by a multi-gene approach proposed by Gallardo and collaborators [45] in order to characterize the epidemiological dynamics of the viral strains circulating in Italy. To reach this goal, 132 ASFV strains ASFV were selected, which represents the largest ASFV partial sequencing effort to date. 

This study produced a total of 792 ASFV partial sequences referring to 132 different samples collected during the epidemic wave of 2022–2023, and these data offer novel insights into the ASF viruses associated with the ASF circulating in Italy. A comprehensive depiction of the genetic variability of ASFV is provided, focusing on nucleotide alterations and small and large insertions/deletions (indels) among the specimens collected in various cases or outbreaks.

The molecular marker variability described cannot be associated with wild and domestic populations.

A phylogenetic analysis of the C-terminal portion of the B646L gene allowed all the isolates from the 2022–2023 Italian epidemic wave to be classified as members of ASFV-p72 genotype II, exhibiting a nucleotide identity exceeding 99.7%. This observation initially implied a temporal and geographical restitution of all the strains, representing all the infected areas.

Despite the apparent temporal stability of the ASFV genomes, this study identified major genetic variants amongst the genotype II ASF viruses from Italy. Based on the concatenated nucleotide sequences of CVR, IGR-ECO1, O174L, K145R, MGF and ECO2, 24 genetic groups have been previously distinguished in Europe so far [45]. 

When investigating the essential genetic markers (CVR, IGR-ECO1, O174L, K145R, MGF and ECO2) identified for differentiating between ASFV genotype II of Italian origins, four genetic groups were identified: 3 and 19, previously described by Gallardo and collaborators [45], and two new putative genetic groups, 25 and 26. 

Genetic group 3 consists of viruses collected initially from the northwestern cluster, and it was discovered in Piedmont, Lombardy, Liguria and Emilia-Romagna. Most ASF cases reported in this area are attributed to wild boar, with outbreaks in domestic pigs only in Lombardy. Subsequently, it was also identified in wild boar in the Campania cluster, despite the geographical distance and the absence of apparent connections. The presence of genetic group 3 was confirmed in Sardinia, where the virus arrived through a long-distance jump transmission. The outbreak was identified in a small pig farm, and the ASF isolates showed a very high identity with the ASFV strains collected in a pig farm located in Zinasco Municipality (Pavia province, Lombardy region, Italy). Genetic group 3 stands out as predominant within the European ASFVs, detected in countries such as Ukraine, Belarus, Lithuania, Poland, Latvia, Estonia, Czech Republic, Romania, Moldova, Hungary, Slovakia and Italy [45].

Furthermore, in the northwestern cluster, in Alessandria province, a point mutation was identified in the O174L marker in four wild boars when compared to Georgia 2007/1, and it was reported as a C–T transition. Based on this SNP, the four strains (8549_2284/AL/2023, 22700_2598/AL/2023, 22700_2645/AL/2023 and 22700_2646/AL/2023) were classified into an additional new genetic cluster, namely, 26 (CVR-I, IGR-ECO1-II, O174L-I-SNP1, K145R-I, MGF-I, ECO-I). This gene encodes the DNA polymerase X (PolX), a well-characterized enzyme involved in base-excision repair [62]. The presence of other SNPs and indels has been described by Raminez-Medina and collaborators [56], but the biological effect has yet to be investigated.

Genetic group 19 was identified in the Lazio region, in the central Italy cluster of infection during 2022, in wild boar cases and one outbreak in domestic pigs. Indeed, in 2023, in the same area, the occurrence of a new variant for the MGF marker led to the definition of a new genetic group: 25 (CVR-I, IGR-ECO1-II, O174L-I, K145R-I, MGF-VIII, ECO-II), so far described only in the municipality of Rome. The observed variability in the MGF marker region is the result of two different TRS sets located in the non-coding intergenic region extending from the MGF 505 9R to 10R genes. The first TRS set, observed in all the isolates in this study, showed a pattern referred to as ABBCD. The second set, found in all the infection clusters, except the one in Lazio, followed the EFGHH pattern, resulting in MGF variant I. The samples from Rome showed temporal variation, with the ASFV strains from 2022 matching those from the rest of Italy, and the samples from 2023 showing a new combination of tandem repeats (ABBCD-EGHH), termed MGF variant VIII. This is the first time that this MGF variant has been described. Nevertheless, when we consulted the nucleotide database of the National Center for Biotechnology Information (NCBI) [63] on April 2024, the loss of repeat F was observed in the Chinese isolate CN/GD/2022 (accession number OR290104.2), but it shows a different molecular marker combination when compared to the genetic group 25 ASFV isolates collected in the Lazio cluster.

Finally, genetic group 19 was identified in the Calabria cluster in both wild boar and domestic pigs. In this area, the epidemiological scenario of ASF was characterized by a series of cases reported mainly in wild boar and simultaneously in domestic pigs (mainly backyard and small farms). Genetic group 19 is the second most represented after genetic group 3 in the European territory, particularly present in Romania, Bulgaria, Serbia, Greece and North Macedonia [45].

This research has presented crucial molecular insights into the evolution of ASFV genotype II and offered the scientific community highly valuable data for the management and control of ASF infection. There is a bias in the available sequences from other globally infected countries and it affects the trace-back and trace-on of ASF genotype II introduction. 

In particular, this research shows a picture of ASF spread in different clusters during the Italian ASF genotype II epidemic wave. Other ASFV-positive samples are under investigation to update the genomic representation of the ASFV genotype II Italian collection.

We have no information about the virulence/transmissibility because most of the samples analyzed come from samples taken for passive surveillance and represented by wild boar carcasses. In the rare cases where the host involved was domestic pig, the classic clinical symptoms have been described. At the moment, some animal experiments are underway to verify the phenotype of the strains with greater genetic variability.

Based on the multi-gene approach, it could be possible to suppose more than a single and independent introduction in Italy. 

Further investigations are ongoing to clarify the spread of ASF on the Italian mainland. This includes whole-genome sequencing and a phylogenetic analysis of the Italian ASF strains from both hosts.

Molecular characterization of the ASF virus through different molecular approaches is extremely important to identify the routes of introduction of the infection. In particular, in a country like Italy, where the infection has been reported in completely distinct geographical areas and also without territorial contiguity, it is important to identify the routes of introduction of the virus. Thanks to this identification, control strategies can be implemented that concern not only the management of the wild boar as a reservoir of the virus, but mainly the passive surveillance activities and the biosecurity measures.

## Figures and Tables

**Table 1:** Summary of the ASFV genotype II isolates from Italian clusters/outbreak analyzed in this study.

Cluster/Outbreak	Region	Provinces	Collecting Date From–To (DD/MM/YYYY)	Host		Total
				Wild Boar	Domestic Pig	
Northwest	Piedmont	Alessandria	07/01/2022–24/02/2023	46		46
	Liguria	Genoa, Savona	13/01/2022–22/12/2022	11		11
	Lombardy	Pavia	16/08/2023–07/09/2023	1	7	8
	Emilia Romagna	Piacenza	16/11/2023	1		1
Lazio	Lazio	Rome	29/04/2022–11/07/2023	28	2	30
Campania–Basilicata	Campania	Salerno	22/05/2023–10/06/2023	9		9
Calabria	Reggio Calabria	Reggio Calabria	03/05/2023–17/07/2023	7	17	24
Sardinia	Sardinia	Nuoro	19/09/2023		3	3
				103	29	132

**Table 2:** Assignment of the analyzed samples to ASFV genetic groups based on marker gene (CVR, IGR-ECO1, O174L, K145R, MGF and ECO2) variants.

Cluster/Outbreak	Provinces	CVR	IGR-ECO1	O174L	K145R	MGF	ECO2	Genetic Group
Northwest	Alessandria	I	II	I (91.3%)	I	I	I	3
				I-SNP1 (8.7%) ^1^				26
	Genoa, Savona	I	II	I	I	I	I	3
	Pavia	I	II	I	I	I	I	3
	Piacenza	I	II	I	I	I	I	3
Lazio	Rome	I	II	I	I	I (60%) ^2^	II	19
						VIII (40%) ^3^		25
Campania–Basilicata	Salerno	I	II	I	I	I	I	3
Calabria	Reggio Calabria	I	II	I	I	I	II	19
Sardinia	Nuoro	I	II	I	I	I	I	3

^1^: Four wild boars collected in 2023; ^2^: Wild boar and domestic pigs collected in 2022; ^3^: Wild boar and domestic pigs collected in 2023.

## Data Availability

The original data presented in the study are openly available from the National Center for Biotechnology Information (NCBI) at https://www.ncbi.nlm.nih.gov.

## Supplementary Materials

The following supporting information can be downloaded at: https://www.mdpi.com/article/10.3390/v16081185/s1, Table S1: ASFV genotype II isolates from Italian clusters used in this study, genetic characterization and GenBank accession numbers. Figure S1: Maximum likelihood (ML) phylogenetic tree on B646L/p72 gene. Figure S2: Northwestern ASF Italian cluster. Geographical distribution and identification of genetic variants of molecular markers: (a) CVR, (b) IGR-ECO1, (c) K145. Figure S3: Lazio ASF Italian cluster. Geographical distribution and identification of genetic variants of molecular markers: (a) CVR, (b) IGR-ECO1, (c) K145. Figure S4: Campania–Basilicata ASF Italian cluster. Geographical distribution and identification of genetic variants of molecular markers: (a) CVR, (b) IGR-ECO1, (c) K145. Figure S5: Calabria ASF Italian cluster. Geographical distribution and identification of genetic variants of molecular markers: (a) CVR, (b) IGR-ECO1, (c) K145. Figure S6: Sardinia ASF Italian outbreak. Geographical distribution and identification of genetic variants of molecular markers: (a) CVR, (b) IGR-ECO1, (c) K145.
