# Supplementary material for: Genetic Characterization of African Swine Fever Italian Clusters in the 2022–2023 Epidemic Wave by a Multi-Gene Approach
Source: Viruses. 2024 Jul 24;16(8):1185. doi: 10.3390/v16081185 (PMC11360507; doi:10.3390/v16081185)
Supplement: Supplementary file 1 [file viruses-16-01185-s001.zip › viruses-3105281-supplementary.pdf]

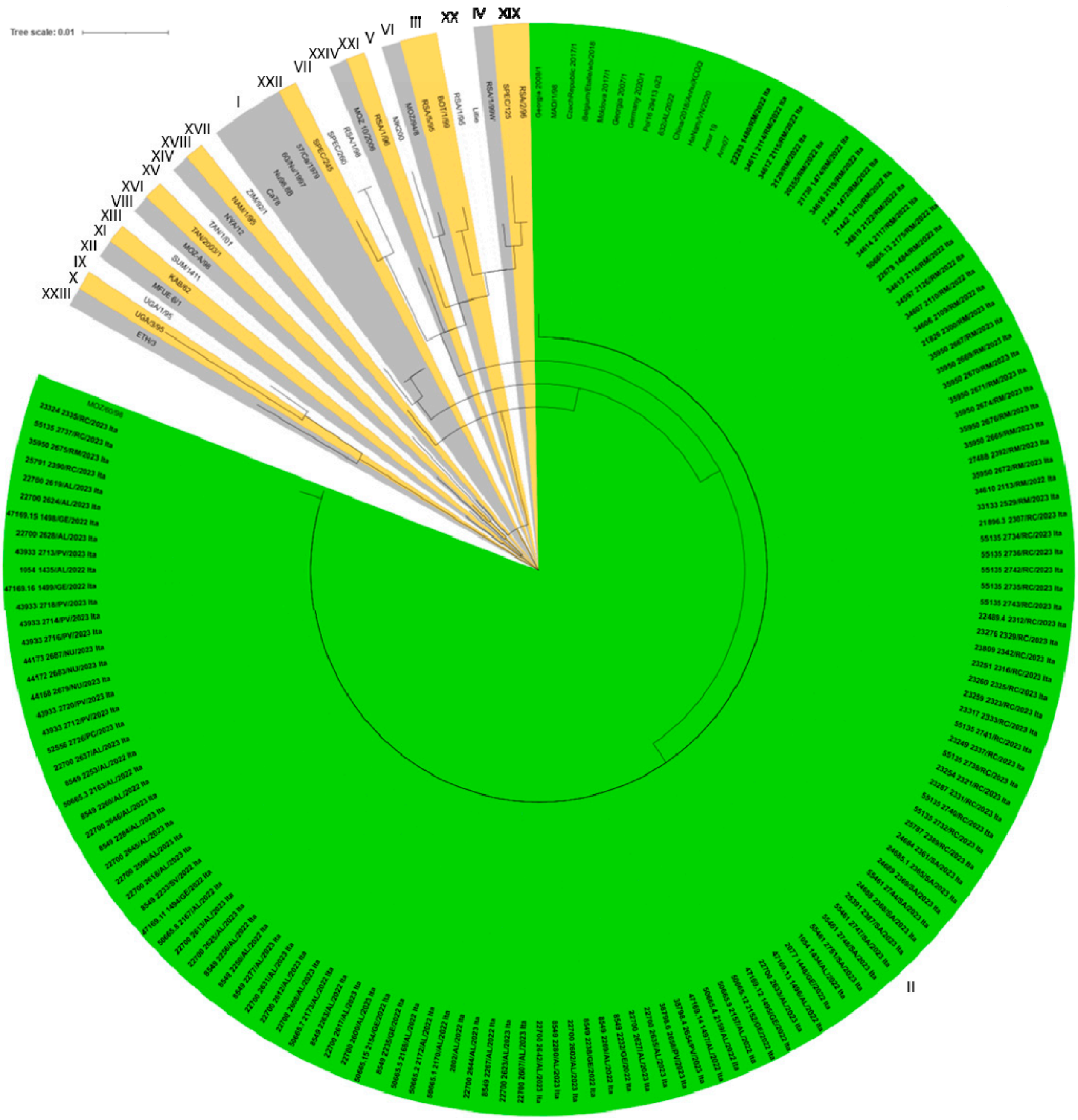

Figure S1: Maximum likelihood (ML) phylogenetic tree based on the alignment of 383 nt from B646L/p72 gene of 176 sequences: 132 analysed in this study (in bold) and 44 reference strains available in GenBank. Bar: number of substitutions per site.

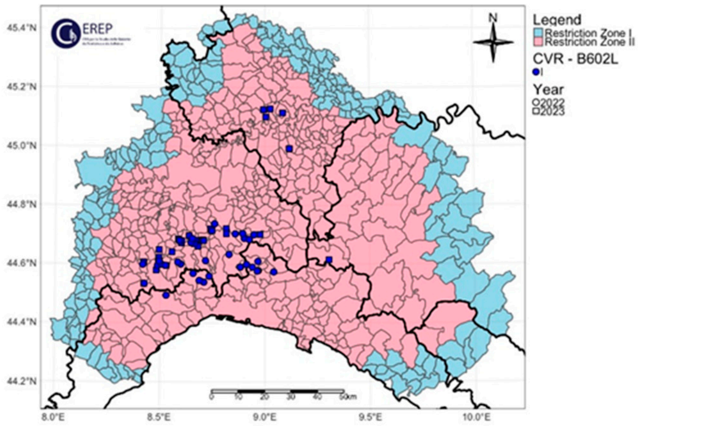

a) CVR

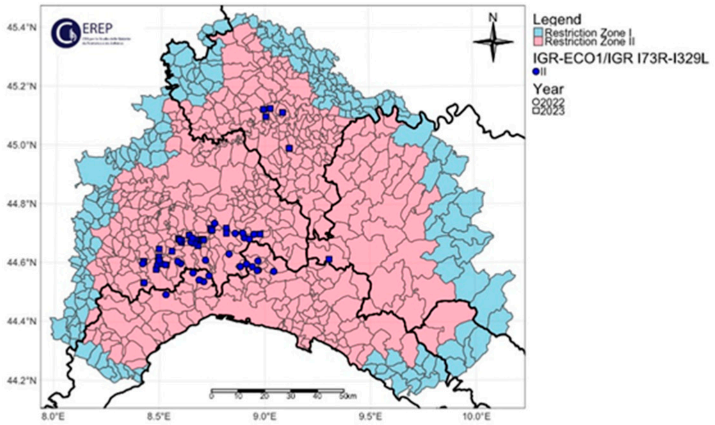

b) IGR-ECO1

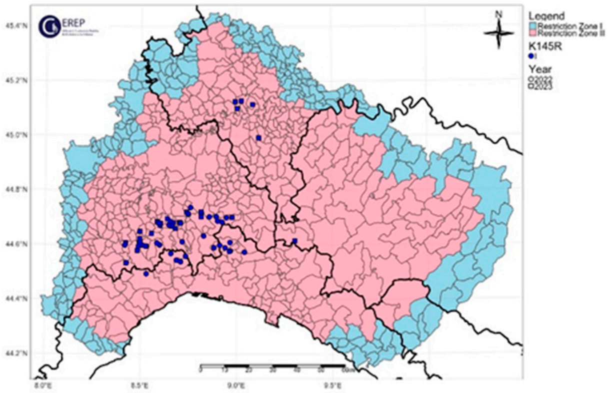

c) K145R

Figure S2: Northwestern ASF Italian cluster: geographical distribution and identification of genetic variants of molecular markers (a) CVR, (b) IGR-ECO1, (c) K145. The *pink* and *light blue* areas represented restriction zone II and restriction zone I, respectively, as to zoning date of 29 April 2024 (according to Commission Implementing Regulation EU 2024/1269). Round shapes indicate year 2022, square shapes year 2023.

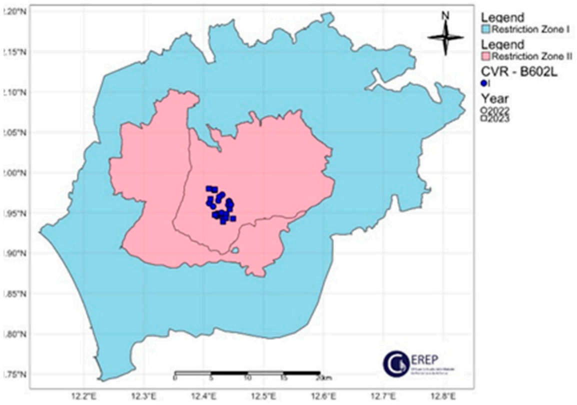

a) CVR

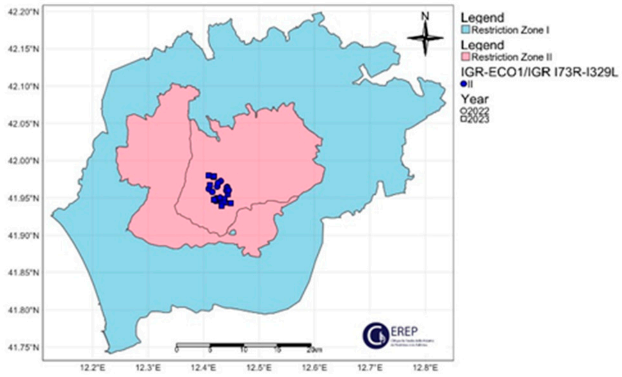

b) IGR-ECO1

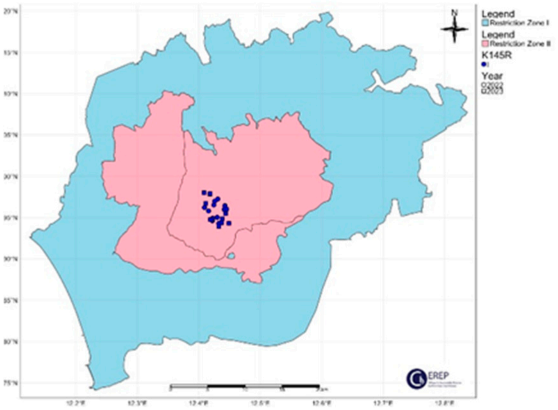

c) K145R

Figure S3: Lazio ASF Italian cluster: geographical distribution and identification of genetic variants of molecular markers (a) CVR, (b) IGR-ECO1, (c) K145. The *pink* and *light blue* areas represented restriction zone II and restriction zone I, respectively, as to zoning date of 29 April 2024 (according to Commission Implementing Regulation EU 2024/1269). Round shapes indicate year 2022, square shapes year 2023.

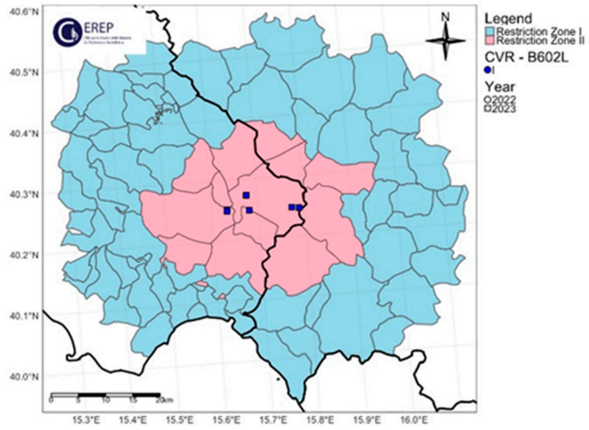

a) CVR

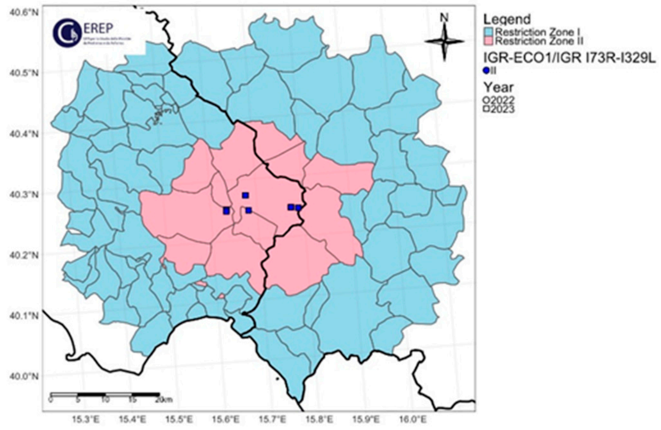

b) IGR-ECO1

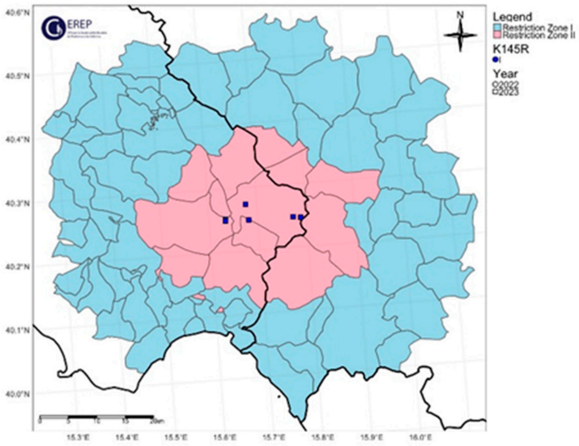

c) K145R

Figure S4: Campania ASF Italian cluster: geographical distribution and identification of genetic variants of molecular markers (a) CVR, (b) IGR-ECO1, (c) K145. The *pink* and *light blue* areas represented restriction zone II and restriction zone I, respectively, as to zoning date of 29 April 2024 (according to Commission Implementing Regulation EU 2024/1269). Round shapes indicate year 2022, square shapes year 2023.

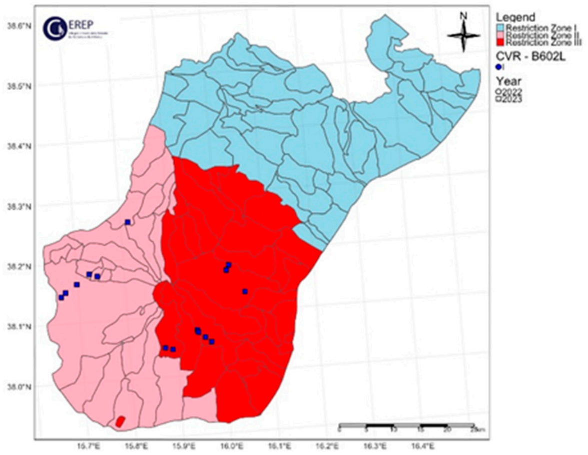

a) CVR

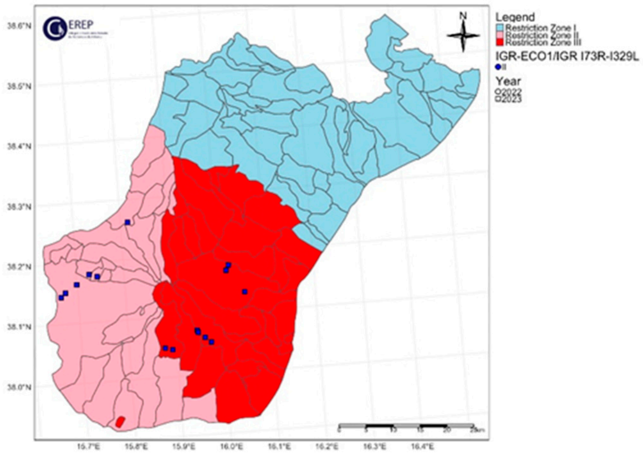

b) IGR-ECO1

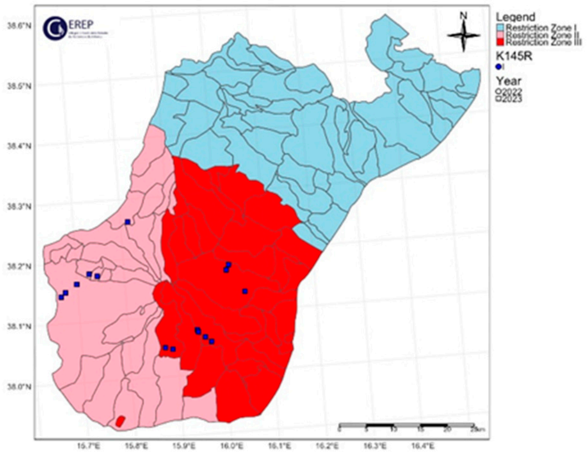

c) K145R

Figure S5: Calabria ASF Italian cluster: geographical distribution and identification of genetic variants of molecular markers (a) CVR, (b) IGR-ECO1, (c) K145. The *red, pink, and light blue* areas represented restriction zone III, restriction zone II and restriction zone I, respectively, as to zoning date of 29 April 2024 (according to Commission Implementing Regulation EU 2024/1269). Round shapes indicate year 2022, square shapes year 2023.

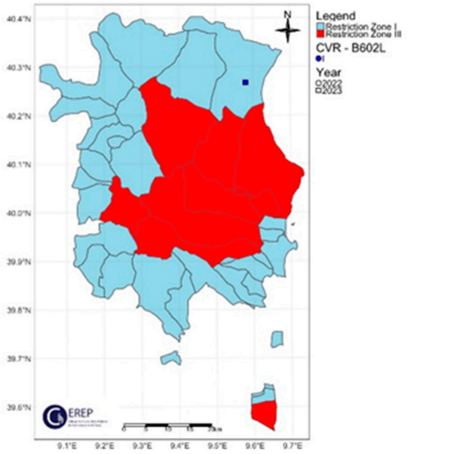

a) CVR

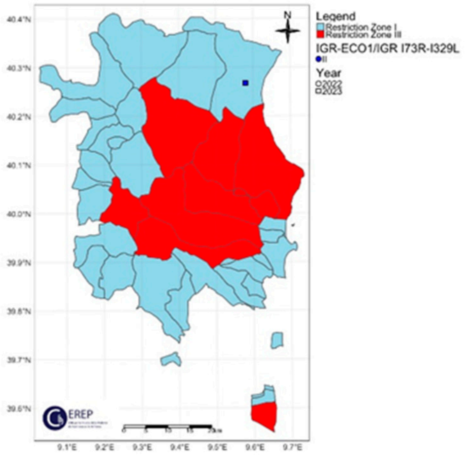

b) IGR-ECO1

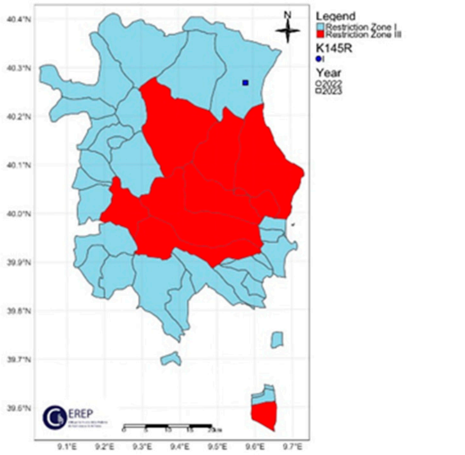

c) K145R

Figure S6: Sardinia ASF Italian outbreak: geographical distribution and identification of genetic variants of molecular markers (a) CVR, (b) IGR-ECO1, (c) K145. The *red* and *light blue* areas represented restriction zone III and restriction zone I, respectively, as to zoning date of 29 April 2024 (according to Commission Implementing Regulation EU 2024/1269). Round shapes indicate year 2022, square shapes year 2023.

#	Isolate name	Region	Province	Collecting date (DD/MM/YYYY)	Host	CVR		IGR-ECO1		O174L		K145R		MGF		ECO2		Genetic Group
						Gene var.	Accession number	Gene var.	Accession number	Gene var.	Accession number	Gene var.	Accession number	Gene var.	Accession number	Gene var.	Accession number	
1	1054_1435/AL/2022	Piedmont	Alessandria	7/1/2022	wild boar	I	PP420224	II	PP420281	I	PP420338	I	PP420395	I	PP420451	I	PP420464	**3**
2	1054_1434/AL/2022	Piedmont	Alessandria	7/1/2022	wild boar	I	PP420223	II	PP420280	I	PP420337	I	PP420394	I	OR966390	I	PP420463	**3**
3	2077_1448/GE/2022	Liguria	Genoa	13/1/2022	wild boar	I	PP420225	II	PP420282	I	PP420339	I	PP420396	I	OR966392	I	PP420465	**3**
4	2802/AL/2022	Piedmont	Alessandria	16/1/2022	wild boar	I	PP420249	II	PP420306	I	PP420363	I	PP420420	I	OR966443	I	PP420489	**3**
5	47169.11_1494/GE/2022	Liguria	Genoa	21/1/2022	wild boar	I	PP420250	II	PP420307	I	PP420364	I	PP420421	I	OR966469	I	PP420490	**3**
6	47169.12_1495/GE/2022	Liguria	Genoa	27/1/2022	wild boar	I	PP420251	II	PP420308	I	PP420365	I	PP420422	I	OR966470	I	PP420491	**3**
7	47169.13_1496/AL/2022	Piedmont	Alessandria	12/3/2022	wild boar	I	PP420252	II	PP420309	I	PP420366	I	PP420423	I	OR966471	I	PP420492	**3**
8	47169.15_1498/GE/2022	Liguria	Genoa	21/3/2022	wild boar	I	PP420254	II	PP420311	I	PP420368	I	PP420425	I	PP420454	I	PP420494	**3**
9	47169.16_1499/GE/2022	Liguria	Genoa	31/3/2022	wild boar	I	PP420255	II	PP420312	I	PP420369	I	PP420426	I	PP420455	I	PP420495	**3**
10	47169.14_1497/AL/2022	Piedmont	Alessandria	2/4/2022	wild boar	I	PP420253	II	PP420310	I	PP420367	I	PP420424	I	PP420453	I	PP420493	**3**
11	50665.4_2159/AL/2022	Piedmont	Alessandria	28/4/2022	wild boar	I	PP420261	II	PP420318	I	PP420375	I	PP420432	I	PP420459	I	PP420501	**3**
12	20355/RM/2022	Lazio	Rome	29/4/2022	wild boar	I	PP420836	II	PP420866	I	PP420896	I	PP420611	I	OR966391	II	PP420573	**19**
13	50665.9_2157/AL/2022	Piedmont	Alessandria	30/4/2022	wild boar	I	PP420265	II	PP420322	I	PP420379	I	PP420436	I	PP420461	I	PP420505	**3**
14	21442_1470/RM/2022	Lazio	Rome	6/5/2022	wild boar	I	PP420838	II	PP420868	I	PP420898	I	PP420613	I	OR966394	II	PP420575	**19**
15	21730_1474/RM/2022	Lazio	Rome	9/5/2022	wild boar	I	PP420840	II	PP420870	I	PP420900	I	PP420615	I	PP420605	II	PP420577	**19**
16	21444_1472/RM/2022	Lazio	Rome	9/5/2022	wild boar	I	PP420839	II	PP420869	I	PP420899	I	PP420614	I	PP420604	II	PP420576	**19**
17	22283_1480/RM/2022	Lazio	Rome	12/5/2022	wild boar	I	PP420842	II	PP420872	I	PP420902	I	PP420617	I	OR966398	II	PP420579	**19**
18	22678_1484/RM/2022	Lazio	Rome	15/5/2022	wild boar	I	PP420843	II	PP420873	I	PP420903	I	PP420618	I	PP420606	II	PP420580	**19**
19	50665.3_2163/AL/2022	Piedmont	Alessandria	23/5/2022	wild boar	I	PP420260	II	PP420317	I	PP420374	I	PP420431	I	PP420458	I	PP420500	**3**
20	50665.8_2167/AL/2022	Piedmont	Alessandria	26/5/2022	wild boar	I	PP420264	II	PP420321	I	PP420378	I	PP420435	I	OR966476	I	PP420504	**3**
21	34606_2109/RM/2022	Lazio	Rome	1/6/2022	wild boar	I	PP420847	II	PP420877	I	PP420907	I	PP420622	I	OR966446	II	PP420584	**19**
22	50665.5_2168/AL/2022	Piedmont	Alessandria	2/6/2022	wild boar	I	PP420262	II	PP420319	I	PP420376	I	PP420433	I	PP420460	I	PP420502	**3**
23	34607_2110/RM/2022	Lazio	Rome	8/6/2022	wild boar	I	PP420848	II	PP420878	I	PP420908	I	PP420623	I	OR966447	II	PP420585	**19**
24	34597_2126/RM/2022	Lazio	Rome	9/6/2022	dom. pig	I	PP420846	II	PP420876	I	PP420906	I	PP420621	I	PP420607	II	PP420583	**19**
25	34610_2113/RM/2022	Lazio	Rome	11/6/2022	wild boar	I	PP420849	II	PP420879	I	PP420909	I	PP420586	I	PP420608	II	PP420624	**19**
26	34611_2114/RM/2022	Lazio	Rome	12/6/2022	wild boar	I	PP420850	II	PP420880	I	PP420910	I	PP420625	I	OR966448	II	PP420587	**19**
27	34612_2115/RM/2022	Lazio	Rome	12/6/2022	wild boar	I	PP420851	II	PP420881	I	PP420911	I	PP420626	I	PP420609	II	PP420588	**19**
28	34613_2116/RM/2022	Lazio	Rome	12/6/2022	wild boar	I	PP420852	II	PP420882	I	PP420912	I	PP420627	I	PP420610	II	PP420589	**19**

29	34614_2117/RM/2022	Lazio	Rome	15/6/2022	wild boar	I	PP420853	II	PP420883	I	PP420913	I	PP420628	I	OR966449	II	PP420590	**19**
30	34616_2119/RM/2022	Lazio	Rome	18/6/2022	wild boar	I	PP420854	II	PP420884	I	PP420914	I	PP420629	I	OR966450	II	PP420591	**19**
31	34619_2122/RM/2022	Lazio	Rome	23/6/2022	wild boar	I	PP420855	II	PP420885	I	PP420915	I	PP420630	I	OR966451	II	PP420592	**19**
32	50665.2_2172/AL/2022	Piedmont	Alessandria	10/7/2022	wild boar	I	PP420259	II	PP420316	I	PP420373	I	PP420430	I	OR966474	I	PP420499	**3**
33	50665.7_2173/AL/2022	Piedmont	Alessandria	10/7/2022	wild boar	I	PP420263	II	PP420320	I	PP420377	I	PP420434	I	OR966475	I	PP420503	**3**
34	50665.12_2152/GE/2022	Liguria	Genoa	25/7/2022	wild boar	I	PP420256	II	PP420313	I	PP420370	I	PP420427	I	PP420456	I	PP420496	**3**
35	50665.1_2170/AL/2022	Piedmont	Alessandria	4/8/2022	wild boar	I	PP420258	II	PP420315	I	PP420372	I	PP420429	I	PP420457	I	PP420498	**3**
36	2129/RM/2022	Lazio	Rome	11/8/2022	wild boar	I	PP420837	II	PP420867	I	PP420897	I	PP420612	I	PP420603	II	PP420574	**19**
37	50665.15_2154/GE/2022	Liguria	Genoa	24/8/2022	wild boar	I	PP420257	II	PP420314	I	PP420371	I	PP420428	I	OR966473	I	PP420497	**3**
38	50665.13_2175/RM/2022	Lazio	Rome	27/8/2022	wild boar	I	PP420865	II	PP420895	I	PP420925	I	PP420640	I	OR966472	II	PP420602	**19**
39	8549_2250/AL/2022	Piedmont	Alessandria	2/12/2022	wild boar	I	PP420270	II	PP420327	I	PP420384	I	PP420441	I	OR966481	I	PP420510	**3**
40	8549_2253/AL/2022	Piedmont	Alessandria	12/12/2022	wild boar	I	PP420271	II	PP420328	I	PP420385	I	PP420442	I	OR966482	I	PP420511	**3**
41	8549_2232/GE/2022	Liguria	Genoa	16/12/2022	wild boar	I	PP420266	II	PP420323	I	PP420380	I	PP420437	I	OR966477	I	PP420506	**3**
42	8549_2233/SV/2022	Liguria	Savona	17/12/2022	wild boar	I	PP420267	II	PP420324	I	PP420381	I	PP420438	I	OR966478	I	PP420507	**3**
43	8549_2256/AL/2022	Piedmont	Alessandria	17/12/2022	wild boar	I	PP420272	II	PP420329	I	PP420386	I	PP420443	I	OR966483	I	PP420512	**3**
44	8549_2235/GE/2022	Liguria	Genoa	20/12/2022	wild boar	I	PP420268	II	PP420325	I	PP420382	I	PP420439	I	OR966479	I	PP420508	**3**
45	8549_2238/GE/2022	Liguria	Genoa	22/12/2022	wild boar	I	PP420269	II	PP420326	I	PP420383	I	PP420440	I	OR966480	I	PP420509	**3**
46	8549_2260/AL/2022	Piedmont	Alessandria	22/12/2022	wild boar	I	PP420273	II	PP420330	I	PP420387	I	PP420444	I	OR966484	I	PP420513	**3**
47	8549_2263/AL/2022	Piedmont	Alessandria	23/12/2022	wild boar	I	PP420274	II	PP420331	I	PP420388	I	PP420445	I	OR966485	I	PP420514	**3**
48	8549_2267/AL/2022	Piedmont	Alessandria	30/12/2022	wild boar	I	PP420275	II	PP420332	I	PP420389	I	PP420446	I	OR966486	I	PP420515	**3**
49	8549_2269/AL/2022	Piedmont	Alessandria	30/12/2022	wild boar	I	PP420276	II	PP420333	I	PP420390	I	PP420447	I	OR966487	I	PP420516	**3**
50	8549_2277/AL/2023	Piedmont	Alessandria	3/1/2023	wild boar	I	PP420277	II	PP420334	I	PP420391	I	PP420448	I	PP420462	I	PP420517	**3**
51	8549_2280/AL/2023	Piedmont	Alessandria	10/1/2023	wild boar	I	PP420278	II	PP420335	I	PP420392	I	PP420449	I	OR966488	I	PP420518	**3**
52	8549_2284/AL/2023	Piedmont	Alessandria	11/1/2023	wild boar	I	PP420279	II	PP420336	I/SNP1	PP420393	I	PP420450	I	OR966489	I	PP420519	**26**
53	22700_2598/AL/2023	Piedmont	Alessandria	14/1/2023	wild boar	I	PP420226	II	PP420283	I/SNP1	PP420340	I	PP420397	I	OR966400	I	PP420466	**26**
54	22700_2600/AL/2023	Piedmont	Alessandria	14/1/2023	wild boar	I	PP420227	II	PP420284	I	PP420341	I	PP420398	I	OR966401	I	PP420467	**3**
55	22700_2602/AL/2023	Piedmont	Alessandria	15/1/2023	wild boar	I	PP420228	II	PP420285	I	PP420342	I	PP420399	I	OR966402	I	PP420468	**3**
56	22700_2607/AL/2023	Piedmont	Alessandria	19/1/2023	wild boar	I	PP420229	II	PP420286	I	PP420343	I	PP420400	I	OR966403	I	PP420469	**3**
57	22700_2608/AL/2023	Piedmont	Alessandria	19/1/2023	wild boar	I	PP420230	II	PP420287	I	PP420344	I	PP420401	I	OR966404	I	PP420470	**3**
58	22700_2613/AL/2023	Piedmont	Alessandria	22/1/2023	wild boar	I	PP420232	II	PP420289	I	PP420346	I	PP420403	I	OR966406	I	PP420472	**3**
59	22700_2612/AL/2023	Piedmont	Alessandria	22/1/2023	wild boar	I	PP420231	II	PP420288	I	PP420345	I	PP420402	I	OR966405	I	PP420471	**3**

60	22700_2617/AL/2023	Piedmont	Alessandria	25/1/2023	wild boar	I	PP420233	II	PP420290	I	PP420347	I	PP420404	I	OR966407	I	PP420473	**3**
61	22700_2618/AL/2023	Piedmont	Alessandria	25/1/2023	wild boar	I	PP420234	II	PP420291	I	PP420348	I	PP420405	I	PP420452	I	PP420474	**3**
62	22700_2624/AL/2023	Piedmont	Alessandria	26/1/2023	wild boar	I	PP420237	II	PP420294	I	PP420351	I	PP420408	I	OR966410	I	PP420477	**3**
63	22700_2619/AL/2023	Piedmont	Alessandria	26/1/2023	wild boar	I	PP420235	II	PP420292	I	PP420349	I	PP420406	I	OR966408	I	PP420475	**3**
64	22700_2623/AL/2023	Piedmont	Alessandria	26/1/2023	wild boar	I	PP420236	II	PP420293	I	PP420350	I	PP420407	I	OR966409	I	PP420476	**3**
65	22700_2627/AL/2023	Piedmont	Alessandria	27/1/2023	wild boar	I	PP420239	II	PP420296	I	PP420353	I	PP420410	I	OR966412	I	PP420479	**3**
66	22700_2628/AL/2023	Piedmont	Alessandria	27/1/2023	wild boar	I	PP420240	II	PP420297	I	PP420354	I	PP420411	I	OR966413	I	PP420480	**3**
67	22700_2625/AL/2023	Piedmont	Alessandria	29/1/2023	wild boar	I	PP420238	II	PP420295	I	PP420352	I	PP420409	I	OR966411	I	PP420478	**3**
68	22700_2631/AL/2023	Piedmont	Alessandria	29/1/2023	wild boar	I	PP420241	II	PP420298	I	PP420355	I	PP420412	I	OR966414	I	PP420481	**3**
69	22700_2637/AL/2023	Piedmont	Alessandria	30/1/2023	wild boar	I	PP420244	II	PP420301	I	PP420358	I	PP420415	I	OR966417	I	PP420484	**3**
70	22700_2633/AL/2023	Piedmont	Alessandria	31/1/2023	wild boar	I	PP420242	II	PP420299	I	PP420356	I	PP420413	I	OR966415	I	PP420482	**3**
71	22700_2635/AL/2023	Piedmont	Alessandria	31/1/2023	wild boar	I	PP420243	II	PP420300	I	PP420357	I	PP420414	I	OR966416	I	PP420483	**3**
72	22700_2642/AL/2023	Piedmont	Alessandria	2/2/2023	wild boar	I	PP420245	II	PP420302	I	PP420359	I	PP420416	I	OR966418	I	PP420485	**3**
73	22700_2644/AL/2023	Piedmont	Alessandria	12/2/2023	wild boar	I	PP420246	II	PP420303	I	PP420360	I	PP420417	I	OR966419	I	PP420486	**3**
74	22700_2645/AL/2023	Piedmont	Alessandria	24/2/2023	wild boar	I	PP420247	II	PP420304	I/SNP1	PP420361	I	PP420418	I	OR966420	I	PP420487	**26**
75	22700_2646/AL/2023	Piedmont	Alessandria	24/2/2023	wild boar	I	PP420248	II	PP420305	I/SNP1	PP420362	I	PP420419	I	OR966421	I	PP420488	**26**
76	21896.3_2307/RC/2023	Calabria	Reggio Calabria	3/5/2023	wild boar	I	PP420641	II	PP420674	I	PP420707	I	PP420740	I	OR966396	II	PP420788	**19**
77	21826_2300/RM/2023	Lazio	Rome	8/5/2023	wild boar	I	PP420841	II	PP420871	I	PP420901	I	PP420616	VIII	OR966395	II	PP420578	**25**
78	22489.4_2312/RC/2023	Calabria	Reggio Calabria	9/5/2023	dom. pig	I	PP420642	II	PP420675	I	PP420708	I	PP420741	I	OR966399	II	PP420789	**19**
79	23251_2316/RC/2023	Calabria	Reggio Calabria	11/5/2023	dom. pig	I	PP420644	II	PP420677	I	PP420710	I	PP420743	I	OR966423	II	PP420791	**19**
80	23260_2325/RC/2023	Calabria	Reggio Calabria	11/5/2023	dom. pig	I	PP420647	II	PP420680	I	PP420713	I	PP420746	I	OR966426	II	PP420794	**19**
81	23276_2329/RC/2023	Calabria	Reggio Calabria	11/5/2023	dom. pig	I	PP420648	II	PP420681	I	PP420714	I	PP420747	I	OR966427	II	PP420795	**19**
82	23254_2321/RC/2023	Calabria	Reggio Calabria	13/5/2023	dom. pig	I	PP420645	II	PP420678	I	PP420711	I	PP420744	I	OR966424	II	PP420792	**19**
83	23259_2323/RC/2023	Calabria	Reggio Calabria	13/5/2023	dom. pig	I	PP420646	II	PP420679	I	PP420712	I	PP420745	I	OR966425	II	PP420793	**19**
84	23249_2337/RC/2023	Calabria	Reggio Calabria	14/5/2023	dom. pig	I	PP420643	II	PP420676	I	PP420709	I	PP420742	I	OR966422	II	PP420790	**19**
85	23324_2335/RC/2023	Calabria	Reggio Calabria	14/5/2023	dom. pig	I	PP420651	II	PP420684	I	PP420717	I	PP420750	I	OR966430	II	PP420798	**19**
86	23287_2331/RC/2023	Calabria	Reggio Calabria	14/5/2023	dom. pig	I	PP420649	II	PP420682	I	PP420715	I	PP420748	I	OR966428	II	PP420796	**19**
87	23317_2333/RC/2023	Calabria	Reggio Calabria	14/5/2023	dom. pig	I	PP420650	II	PP420683	I	PP420716	I	PP420749	I	OR966429	II	PP420797	**19**
88	23809_2342/RC/2023	Calabria	Reggio Calabria	16/5/2023	dom. pig	I	PP420652	II	PP420685	I	PP420718	I	PP420751	I	OR966431	II	PP420799	**19**

89	24685.1_2365/SA/2023	Campania	Salerno	22/5/2023	wild boar	I	PP420654	II	PP420687	I	PP420720	I	PP420753	I	OR966435	I	PP420801	**3**
90	24689_2369/SA/2023	Campania	Salerno	22/5/2023	wild boar	I	PP420656	II	PP420689	I	PP420722	I	PP420755	I	OR966437	I	PP420803	**3**
91	24684_2361/SA/2023	Campania	Salerno	22/5/2023	wild boar	I	PP420653	II	PP420686	I	PP420719	I	PP420752	I	OR966434	I	PP420800	**3**
92	24688_2368/SA/2023	Campania	Salerno	22/5/2023	wild boar	I	PP420655	II	PP420688	I	PP420721	I	PP420754	I	OR966436	I	PP420802	**3**
93	35950_2665/RM/2023	Lazio	Rome	26/5/2023	wild boar	I	PP420856	II	PP420886	I	PP420916	I	PP420631	VIII	OR966453	II	PP420593	**25**
94	25787_2389/RC/2023	Calabria	Reggio Calabria	28/5/2023	wild boar	I	PP420658	II	PP420691	I	PP420724	I	PP420757	I	OR966440	II	PP420805	**19**
95	55461_2744/SA/2023	Campania	Salerno	28/5/2023	wild boar	I	PP420670	II	PP420703	I	PP420736	I	PP420769	I	PP420784	I	PP420817	**3**
96	25391_2387/SA/2023	Campania	Salerno	29/5/2023	wild boar	I	PP420657	II	PP420690	I	PP420723	I	PP420756	I	OR966439	I	PP420804	**3**
97	25791_2390/RC/2023	Calabria	Reggio Calabria	29/5/2023	wild boar	I	PP420659	II	PP420692	I	PP420725	I	PP420758	I	OR966441	II	PP420806	**19**
98	55461_2747/SA/2023	Campania	Salerno	31/5/2023	wild boar	I	PP420671	II	PP420704	I	PP420737	I	PP420770	I	PP420785	I	PP420818	**3**
99	55461_2748/SA/2023	Campania	Salerno	4/6/2023	wild boar	I	PP420672	II	PP420705	I	PP420738	I	PP420771	I	PP420786	I	PP420819	**3**
100	27488_2392/RM/2023	Lazio	Rome	7/6/2023	wild boar	I	PP420844	II	PP420874	I	PP420904	I	PP420619	VIII	OR966442	II	PP420581	**25**
101	35950_2667/RM/2023	Lazio	Rome	10/6/2023	wild boar	I	PP420857	II	PP420887	I	PP420917	I	PP420632	VIII	OR966454	II	PP420594	**25**
102	55461_2751/SA/2023	Campania	Salerno	10/6/2023	wild boar	I	PP420673	II	PP420706	I	PP420739	I	PP420772	I	PP420787	I	PP420820	**3**
103	35950_2670/RM/2023	Lazio	Rome	13/6/2023	wild boar	I	PP420859	II	PP420889	I	PP420919	I	PP420634	VIII	OR966456	II	PP420596	**25**
104	35950_2669/RM/2023	Lazio	Rome	14/6/2023	wild boar	I	PP420858	II	PP420888	I	PP420918	I	PP420633	VIII	OR966455	II	PP420595	**25**
105	35950_2671/RM/2023	Lazio	Rome	14/6/2023	wild boar	I	PP420860	II	PP420890	I	PP420920	I	PP420635	VIII	OR966457	II	PP420597	**25**
106	35950_2672/RM/2023	Lazio	Rome	14/6/2023	wild boar	I	PP420861	II	PP420891	I	PP420921	I	PP420636	VIII	OR966458	II	PP420598	**25**
107	55135_2734/RC/2023	Calabria	Reggio Calabria	16/6/2023	dom. pig	I	PP420661	II	PP420694	I	PP420727	I	PP420760	I	PP420775	II	PP420808	**19**
108	35950_2674/RM/2023	Lazio	Rome	17/6/2023	wild boar	I	PP420862	II	PP420892	I	PP420922	I	PP420637	VIII	OR966459	II	PP420599	**25**
109	55135_2735/RC/2023	Calabria	Reggio Calabria	18/6/2023	dom. pig	I	PP420662	II	PP420695	I	PP420728	I	PP420761	I	PP420776	II	PP420809	**19**
110	55135_2736/RC/2023	Calabria	Reggio Calabria	18/6/2023	dom. pig	I	PP420663	II	PP420696	I	PP420729	I	PP420762	I	PP420777	II	PP420810	**19**
111	55135_2743/RC/2023	Calabria	Reggio Calabria	20/6/2023	dom. pig	I	PP420669	II	PP420702	I	PP420735	I	PP420768	I	PP420783	II	PP420816	**19**
112	55135_2732/RC/2023	Calabria	Reggio Calabria	23/6/2023	wild boar	I	PP420660	II	PP420693	I	PP420726	I	PP420759	I	PP420773	II	PP420807	**19**
113	55135_2737/RC/2023	Calabria	Reggio Calabria	26/6/2023	wild boar	I	PP420664	II	PP420697	I	PP420730	I	PP420763	I	PP420778	II	PP420811	**19**
114	35950_2675/RM/2023	Lazio	Rome	27/6/2023	wild boar	I	PP420863	II	PP420893	I	PP420923	I	PP420638	VIII	OR966460	II	PP420600	**25**
115	55135_2738/RC/2023	Calabria	Reggio Calabria	27/6/2023	wild boar	I	PP420665	II	PP420698	I	PP420731	I	PP420764	I	PP420779	II	PP420812	**19**
116	35950_2676/RM/2023	Lazio	Rome	28/6/2023	wild boar	I	PP420864	II	PP420894	I	PP420924	I	PP420639	VIII	OR966461	II	PP420601	**25**
117	55135_2740/RC/2023	Calabria	Reggio Calabria	28/6/2023	dom. pig	I	PP420666	II	PP420699	I	PP420732	I	PP420765	I	PP420780	II	PP420813	**19**

118	55135_2741/RC/2023	Calabria	Reggio Calabria	28/6/2023	wild boar	I	PP420667	II	PP420700	I	PP420733	I	PP420766	I	PP420781	II	PP420814	**19**
119	33133_2529/RM/2023	Lazio	Rome	11/7/2023	dom. pig	I	PP420845	II	PP420875	I	PP420905	I	PP420620	VIII	OR966445	II	PP420582	**25**
120	55135_2742/RC/2023	Calabria	Reggio Calabria	17/7/2023	dom. pig	I	PP420668	II	PP420701	I	PP420734	I	PP420767	I	PP420782	II	PP420815	**19**
121	38798.4_2654/PV/2023	Lombardia	Pavia	16/8/2023	dom. pig	I	PP420520	II	PP420529	I	PP420538	I	PP420547	I	OR966462	I	PP420564	**3**
122	38798.6_2658/PV/2023	Lombardia	Pavia	16/8/2023	dom. pig	I	PP420521	II	PP420530	I	PP420539	I	PP420548	I	OR966463	I	PP420565	**3**
123	43933_2712/PV/2023	Lombardia	Pavia	27/8/2023	wild boar	I	PP420522	II	PP420531	I	PP420540	I	PP420549	I	OR966464	I	PP420566	**3**
124	43933_2713/PV/2023	Lombardia	Pavia	27/8/2023	dom. pig	I	PP420523	II	PP420532	I	PP420541	I	PP420550	I	PP420556	I	PP420567	**3**
125	43933_2714/PV/2023	Lombardia	Pavia	27/8/2023	dom. pig	I	PP420524	II	PP420533	I	PP420542	I	PP420551	I	PP420557	I	PP420568	**3**
126	43933_2716/PV/2023	Lombardia	Pavia	30/8/2023	dom. pig	I	PP420525	II	PP420534	I	PP420543	I	PP420552	I	PP420559	I	PP420569	**3**
127	43933_2718/PV/2023	Lombardia	Pavia	2/9/2023	dom. pig	I	PP420526	II	PP420535	I	PP420544	I	PP420553	I	PP420561	I	PP420570	**3**
128	43933_2720/PV/2023	Lombardia	Pavia	7/9/2023	dom. pig	I	PP420527	II	PP420536	I	PP420545	I	PP420554	I	OR966465	I	PP420571	**3**
129	44168_2679/NU/2023	Sardegna	Nuoro	19/9/2023	dom. pig	I	PP420821	II	PP420824	I	PP420827	I	PP420830	I	OR966466	I	PP420833	**3**
130	44172_2683/NU/2023	Sardegna	Nuoro	19/9/2023	dom. pig	I	PP420822	II	PP420825	I	PP420828	I	PP420831	I	OR966467	I	PP420834	**3**
131	44173_2687/NU/2023	Sardegna	Nuoro	19/9/2023	dom. pig	I	PP420823	II	PP420826	I	PP420829	I	PP420832	I	OR966468	I	PP420835	**3**
132	52556_2726/PC/2023	Emilia Romagna	Piacenza	16/11/2023	wild boar	I	PP420528	II	PP420537	I	PP420546	I	PP420555	I	PP420563	I	PP420572	**3**

Table S1: ASFV genotype II isolates from Italian clusters used in this study, genetic characterization and GenBank accession numbers.
